# Targeting of lactate dehydrogenase C dysregulates the cell cycle and sensitizes breast cancer cells to DNA damage response targeted therapy

**DOI:** 10.1002/1878-0261.13024

**Published:** 2021-06-13

**Authors:** Adviti Naik, Julie Decock

**Affiliations:** ^1^ Translational Cancer and Immunity Center Qatar Biomedical Research Institute (QBRI), Hamad Bin Khalifa University (HBKU) Qatar Foundation (QF) Doha Qatar

**Keywords:** cancer testis antigen, cisplatin, DNA damage, LDHC, olaparib

## Abstract

The cancer testis antigen (CTA) lactate dehydrogenase C (LDHC) is a promising anticancer target with tumor‐specific expression and immunogenicity. Interrogation of breast cancer patient cohorts from The Cancer Genome Atlas (TCGA) and Molecular Taxonomy of Breast Cancer International Consortium (METABRIC) indicate that upregulation of *LDHC* expression correlates with unfavorable prognosis. Although the role of LDHC is well characterized in spermatocytes, its role in tumors remains largely unknown. We investigated whether LDHC is involved in regulating genomic stability and whether it could be targeted to affect tumor cellular fitness. Silencing *LDHC* in four breast cancer cell lines significantly increased the presence of giant cells, nuclear aberrations, DNA damage, and apoptosis. *LDHC‐*silenced cells demonstrated aberrant cell cycle progression with differential expression of cell cycle checkpoint and DNA damage response regulators. In addition, *LDHC* silencing‐induced microtubule destabilization, culminating in increased mitotic catastrophe and reduced long‐term survival. Notably, the clonogenicity of *LDHC‐*silenced cells was further reduced by treatment with the poly (ADP‐ribose) polymerase (PARP) inhibitor olaparib and with the DNA‐damaging drug cisplatin. This study supports the therapeutic potential of targeting LDHC to mitigate cancer cell survival and improve sensitivity to agents that cause DNA damage or inhibit its repair.

## Graphical abstract

LDHC is a lucrative anticancer target given its restricted expression in normal tissues and reexpression in tumors, and its immunogenic and oncogenic traits. Here, we show that targeting LDHC in breast cancer cells dysregulates the cell cycle with increased DNA damage, compromising long‐term cell survival. Moreover, we demonstrate that targeting LDHC augments the cytotoxic activity of DNA damage repair targeted therapy and hence may offer a novel anti‐cancer therapeutic strategy with minimal off‐target effects.

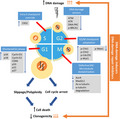

**Abbreviations**
ANOVA: analysis of variance
ATM: ataxia‐telangiectasia mutated
ATR: ataxia telangiectasia and Rad3 related
CDK: cyclin‐dependent kinase
CTA: cancer testis antigen
DDR: DNA damage response
DNA‐PKcs: DNA‐dependent protein kinase, catalytic subunit
EdU: 5‐Ethynyl‐2´‐deoxyuridine
ER: estrogen receptor
GO: Gene Ontology
Her2: human epidermal growth factor receptor 2
HR: homologous recombination
LDH: lactate dehydrogenase
MAP: microtubule‐associated protein
MN: micronuclei
MNC: multi‐nucleated cells
NBUD: nuclear budding
NHEJ: nonhomologous end joining
NPB: nucleoplasmic bridges
PARP: poly ADP ribose polymerase
PI: propidium iodide
PR: progesterone receptor
Rb: retinoblastoma protein
SAC: spindle assembly checkpoint
SEM: standard error of mean
TCGA: The Cancer Genome Atlas
TNBC: triple‐negative breast cancer

## 1 Introduction

Breast cancer remains the most common cancer in women and has now overtaken lung cancer as the leading cause of cancer‐related death in women [1]. The clinical and molecular heterogeneity of breast cancer has led to the definition of numerous subtypes. The immunohistochemically‐defined triple‐negative breast cancer (TNBC) subtype is an aggressive subgroup of breast tumors with high early recurrence rates and poor clinical outcome. Furthermore, TNBC patients do not benefit from targeted therapy due to the absence of estrogen receptor (ER), progesterone receptor (PR), and human epidermal growth factor receptor 2 (HER2) receptor expression. More recently, gene expression profiling identified intrinsic subtypes with improved prognostic stratification, which are independent of standard clinicopathological variables [2, 3]. Among these subtypes, basal‐like breast cancer is associated with the worst prognosis [4]. Triple‐negative and basal‐like breast cancer have a high degree of overlap with TNBCs accounting for about 80% of the latter and being associated with worse clinical outcome compared to nonbasal‐like TNBCs [5, 6, 7, 8]. To date, numerous efforts have been made toward understanding the molecular underpinnings of basal‐like breast cancer with the goal to identify novel and selective targets.

Cancer testis antigens (CTAs) constitute a group of proteins that are endogenously expressed in human germ cells and placental tissue and are re‐expressed in numerous cancer types [9]. Lactate dehydrogenase C (LDHC) is a CTA that belongs to the lactate dehydrogenase (LDH) family of isozymes, comprising of LDHA, LDHB, and LDHC [10]. LDHA and LDHB are the predominant LDH isozymes consisting of four LDH‐M and LDH‐H subunits, respectively, encoded by the *LDHA* and *LDHB* genes and are expressed in the skeletal muscle and heart.

In addition, hetero‐tetramers of the LDH‐M and LDH‐H subunits result in three, albeit less abundant, isoforms with distinct tissue distribution. Of note, LDHC is a testis‐specific LDH that is composed of four LDH‐C subunits. Substrate specificity of each LDH depends on its kinetic properties, with LDHA and LDHC preferentially converting pyruvate to lactate, whereas the reverse reaction is catalyzed by LDHB. LDHC is uniquely positioned as a potential anticancer target thanks to its restricted expression in normal somatic tissues and re‐expression in many tumors [11]. It has a well‐established function in energy metabolism governing sperm motility and male fertility [12, 13]; however, its role in cancer is less understood.

Cui *et al*. [14] recently reported increased LDHC expression in tumor tissue and serum‐derived exosomes of breast cancer patients, which correlated with poor survival, larger tumor size, and recurrence. LDHC expression has also been positively correlated with shorter progression‐free survival in renal cell carcinoma [15]. Moreover, we recently demonstrated that LDHC is an immunogenic tumor‐associated antigen that can elicit a cytotoxic immune response against breast cancer cells [16]. One study demonstrated the presence of tumor‐specific LDHC isoforms with defects in the structure of the catalytic domain that may result into nonfunctional, truncated splice variants [11]. Others have reported that LDHC promotes cancer cell proliferation, migration, and invasion in numerous cancer types [15, 17, 18]. Given the role of a number of CTAs in maintaining genomic integrity during meiosis [19] and in particular of SSX2, NXF2, and FMR1NB in cancer genomic instability [9], we investigated whether LDHC tumor re‐expression might be related to the latter. We focused our study on basal‐like breast cancer which is characterized by increased genomic instability [20, 21, 22]. Using multiple breast cancer cell line models, we show that LDHC is involved in maintaining mitotic fidelity and genomic integrity and that targeting of LDHC improves treatment response to DNA damage response targeted therapy using DNA damage inducers and DNA repair inhibiting agents.

## 2 Materials and methods

### 2.1 Transcriptomic and survival analysis

The Gene Expression Profiling Interactive Analysis (GEPIA) platform (http://gepia.cancer‐pku.cn/index.html) was used to visualize *LDHC* transcriptomic data in normal and breast cancer tissue from The Cancer Genome Atlas (TCGA) and the Genotype‐Tissue Expression (GTEx) datasets. The expression of *LDHC* was calculated in transcripts per million mapped reads (TPM), log‐transformed for differential analysis, and represented by the log2 fold change (log2FC) between tumor and matched normal data (median value of tumor samples minus median value of normal samples). The Human Protein Atlas (HPA) database (https://www.proteinatlas.org/) was used to visualize LDHC RNA and protein expression across multiple normal tissues. The Breast Cancer Integrated Platform (BCIP) (http://www.omicsnet.org/bcancer/database) [23] was utilized to visualize *LDHC* expression in the Molecular Taxonomy of Breast Cancer International Consortium (METABRIC) intrinsic breast cancer subtypes and to generate Kaplan–Meier survival curves stratified by *LDHC* high vs low expression (cutoff = median log2 value).

### 2.2 Cell culture

All breast cancer cell lines were acquired from the American Tissue Culture Collection (ATCC) and authenticated by Short Tandem Repeat (STR) analysis. MDA‐MB‐468 and MDA‐MB‐231 cells were cultured in Dulbecco’s Minimum Essential Media (Gibco) supplemented with 10% v/v fetal bovine serum (FBS) (HyClone US origin, GE Life Sciences), 50 U·mL^−1^ Penicillin and 50 µg·mL^−1^ Streptomycin (Gibco) [24]. BT‐549 cells were cultures in ATCC‐formulated Roswell Park Memorial Institute (RPMI) 1640 medium (Gibco) supplemented with 10% (v/v) FBS (HyClone US origin, GE Lifescience), 50 U·mL^−1^ penicillin and 50 µg·mL^−1^ streptomycin (Gibco), and 0.023 IU·mL^−1^ insulin (Sigma‐Aldrich) [24]. HCC‐1500 cells were cultured in RPMI 1640 media (Gibco) supplemented with 10% FBS, 50 U·mL^−1^ Penicillin and 50 µg·mL^−1^ Streptomycin (Gibco). All cell lines were maintained at 37 °C and 5% CO_2_ in a humidified incubator. Regular mycoplasma testing was conducted using a polymerase chain reaction (PCR)‐based detection assay. Early passage cells (< P10) were used for all experiments.

### 2.3 Stable silencing of *LDHC*

All cell lines were transduced at 70–80% confluency with two purified LDHC‐specific shRNA lentiviral particles (SMARTvector Lentiviral Human LDHC hCMV‐TurboGFP shRNA, Dharmacon, V3SH11240‐227292996 for MDA‐MB‐468 and MDA‐MB‐231 or V3SH11240‐229943916 for BT‐549 and HCC‐1500) or scrambled negative control lentiviral particles (SMARTvector Non‐targeting hCMV‐TurboGFP Control Particles, Dharmacon, #S‐005000‐01) and polybrene transfection reagent (Millipore, Burlington, MA, USA). The packaged viral vectors encode green fluorescent protein (GFP) reporters and a gene conferring resistance to Puromycin antibiotic. Six‐day post‐transduction, transduced cells were selected in complete growth media, supplemented with 0.5 µg·mL^−1^ Puromycin (Sigma‐Aldrich).

### 2.4 Quantitative real‐time PCR

Total RNA was extracted using Tri reagent (Ambion) from cell lines as described previously [25]. Human normal kidney, liver, brain, lung, heart, testis, and thymus total RNA was purchased from Thermo Fisher Scientific (Waltham, MA, USA). Reverse transcription of RNA was performed using MMLV‐Superscript (Thermo Fisher Scientific) and random hexamers. *LDHC* expression was quantified by specific 5′FAM‐3′MGB TaqMan gene expression primer/probe sets (Hs00255650_m1, Applied Biosystems, Foster City, CA, USA). *MAP1B* expression was quantified using primers for SYBR‐based qPCR (F: CACCTCGCCTAGCCTGTC, R: CGGATTCCGAGCTCGATG), designed using PrimerBLAST (NCBI), and PowerUp SYBR Green master mix (Applied Biosystems). qRT‐PCR was performed on the QuantStudio 7 system (Applied Biosystems). Relative expression levels were normalized to the housekeeping gene *RPLPO* (TaqMan primer/probe 4333761F or SYBR primers F: TCCTCGTGGAAGTGACATCG, R: TGGATGATCTTAAGGAAGTAGTTGG).

### 2.5 Immunoblotting

Cell protein lysate was isolated using RIPA buffer (Pierce, Waltham, MA, USA) supplemented with HALT protease and phosphatase inhibitor cocktail (Thermo Fisher Scientific). Western blotting was performed using a standard protocol as previously described [24]. Primary antibodies utilized are listed in Table S1. Horseradish Peroxidase (HRP)‐linked anti‐rabbit/mouse secondary antibody incubation followed by enhanced chemiluminescent substrate (ECL) SuperSignal West Femto (Pierce) incubation was used to visualize the protein bands of interest on the ChemiDoc XRS+ Imaging system (Bio‐Rad, Hercules, CA, USA). Images acquisition and densitometry analysis were performed using the image lab software (Bio‐Rad).

### 2.6 Immunofluorescence microscopy

Immunofluorescence staining was performed according to standard protocols as described previously [24]. Briefly, 80 000 cells were plated on Poly‐Lysine coated glass coverslips (Corning, Corning, NY, USA), fixed with 4% paraformaldehyde (ChemCruz, Dallas, TX, USA), and permeabilized with 0.1% Triton X‐100 (Sigma). Primary antibodies against β‐tubulin (Cell Signaling, Danvers, MA, USA), phospho‐γH2AX (Abcam, Cambridge, UK), alpha‐tubulin (Li‐Cor, Lincoln, NE, USA), acetylated alpha‐tubulin (Cell Signaling), MAP1B (Abcam), BubR1 (Abcam), and Alexa Fluor 568 phalloidin (Thermo Fisher Scientific) are listed in Table S1. Fluorescently‐labeled secondary antibody anti‐rabbit/mouse Alexa Fluor 555/647 (Thermo Fisher Scientific) were used. Cell nuclei were counterstained with 4′,6‐diamidino‐2‐phenylindole (DAPI) (Thermo Fisher Scientific), and cells were mounted using ProLong Gold Antifade reagent (Invitrogen, Waltham, MA, USA). Images were captured using an upright fluorescent microscope (Zeiss Axio Imager, 40× or 100× oil objective) and the Zen Pro 2 acquisition and analyses software. The frequency of nuclear aberrations in cells was determined by manual quantification (100× magnification) of 600 DAPI‐stained nuclei per condition (approximately 120 exclusive fields), and BubR1‐staining was assessed in 90 DAPI‐stained nuclei for each condition (approximately 15 exclusive fields).

### 2.7 Flow cytometry

Cells were harvested by centrifugation and washed in cold phosphate‐buffered saline (PBS). For cell cycle analysis and enumeration of polyploid cells, cells were fixed with ice‐cold 66% ethanol overnight at 4 °C and stained with propidium iodide (PI)/RNase A (Abcam) for 30 min at 37 °C in the dark. For Annexin V/PI quantification, cell were resuspended in 1× Annexin binding buffer (Thermo Fisher Scientific) and stained with Annexin V BV421 (BD Biosciences, Franklin Lakes, NJ, USA) and PI at room temperature for 15 min. Flow cytometry analyses were performed using the LSRFortessa X‐20 system and flowjo software (BD Biosciences).

### 2.8 EdU incorporation assay

Cells were seeded in six‐well plates and incubated for 6 h with 10 µm of the thymidine analogue 5‐Ethynyl‐2´‐deoxyuridine (EdU) in complete growth media. Next, cells were centrifuged, washed twice with PBS, and fixed with ice‐cold 66% ethanol overnight at 4 °C. The fixed cells were brought to room temperature, washed twice with PBS, and stained with the Click‐IT^®^ EdU Alexa Fluor^®^ 647 Flow Cytometry kit as per manufacturer’s instructions (Applied Biosystems). Cells were finally stained with PI and analyzed by flow cytometry.

### 2.9 Cell cycle synchronization

Cells were seeded in a six‐well plate and synchronized at G1/S phase by a double thymidine block [26]. Briefly, cells were first treated with 2 mm thymidine (Sigma‐Aldrich) for 18 h, followed by two washes with PBS and a 9 h release in normal growth media. The cells were then treated with a second thymidine block (2 mm) for 16 h. Subsequently, cells were washed twice with PBS and incubated in complete growth media. Cells were harvested at multiple time points for cell cycle analysis (PI flow cytometry) and western blot analysis.

### 2.10 Clonogenic assay

A total of 10^3^ cells per well were seeded in six‐well plates and maintained in complete growth media for 14 days after which the cells were washed with PBS and stained with crystal violet (5% Bromophenol blue, 25% methanol) for 20 min at 37 °C, as described previously [27]. Excess stain was washed away with distilled water. Stained colonies were first counted manually and then the stain within the colonies was solubilized using 10% sodium dodecyl sulfate (SDS), followed by measurement of the optical density at wavelength 590 nm.

### 2.11 Senescence assay

A total of 5 × 10^5^ cells per well were seeded in six‐well plates and stained for senescence‐associated ß‐galactosidase (SA‐ß‐Gal) activity using the cellular senescence assay kit (Cell Biolabs, Inc., San Diego, CA, USA) according to manufacturer’s instructions. Cells were incubated with staining solution overnight (MDA‐MB‐468, BT‐549, MDA‐MB‐231) or for 4 h (HCC‐1500) and visualized using an inverted light microscope (Zeiss Primo Vert, 40× objective, Oberkochen, Germany) and the zen pro 2 (Oberkochen, Germany) acquisition and analysis software.

### 2.12 EMT RT2 Profiler™ PCR array

Differential expression of 84 cell cycle‐associated genes was determined using the EMT RT2 profiler qPCR Human Cell Cycle array (Qiagen, PAHS‐020Z, Hilden, Germany) according to manufacturer’s guidelines and analyzed using the Qiagen online Data Analysis Tool. Gene expression was normalized to the housekeeping gene *RPLPO*. The threshold for differential expression between shCTRL and shLDHC cells was set at absolute log2 fold change ≥ 2.

### 2.13 Gene Ontology enrichment analysis

Enrichment analysis of gene ontology (GO) was performed using the PANTHER (Protein Analysis Through Evolutionary Relationships) online tool (http://www.pantherdb.org) [28]. Fisher’s exact test with Bonferroni correction was used to identify the enriched GO biological process with *P* ≤ 0.05 and fold enrichment > 50%.

### 2.14 Treatment with DNA‐damaging agents

Cells were seeded in six‐well plates and treated with cisplatin (Sigma‐Aldrich) or olaparib (Selleck Chemicals, Houston, TX, USA). The drug IC50 dosages for each cell line were identified from the Genomics in Drug Sensitivity in Cancer online database (https://www.cancerrxgene.org/) [29] and verified in‐house using the WST‐1 assay (Abcam) after 72‐h treatment with the drug. The IC50 drug dosages used for the various cell lines were as following: 30 µm olaparib and 4 µm cisplatin for MDA‐MB‐468, 210 µm olaparib and 10 µm cisplatin for BT‐549, 28.8 µm olaparib and 19 µm cisplatin for MDA‐MB‐231, and 111 µm olaparib and 140 µm cisplatin for HCC‐1500. Cells were treated for 72 h in complete growth media followed by western blot analyses, clonogenic assay, Annexin V/PI flow cytometry, or immunofluorescence microscopy.

### 2.15 Statistical analysis

Normality of data was assessed using the Shapiro–Wilk test. Nonparametric analyses were conducted using Kruskal–Wallis test, while parametric analyses were performed using Student’s *t*‐test or analysis of variance (ANOVA). *P* value ≤ 0.05 was defined as statistically significant. Data are represented as mean ± standard error of mean (SEM) of at least three independent biological replicates. Statistical analyses and data representation were performed using graphpad prism v8.0.0 (San Diego, CA, USA).

## 3 Results

### 3.1 LDHC expression in breast cancer

**Fig. 1:**
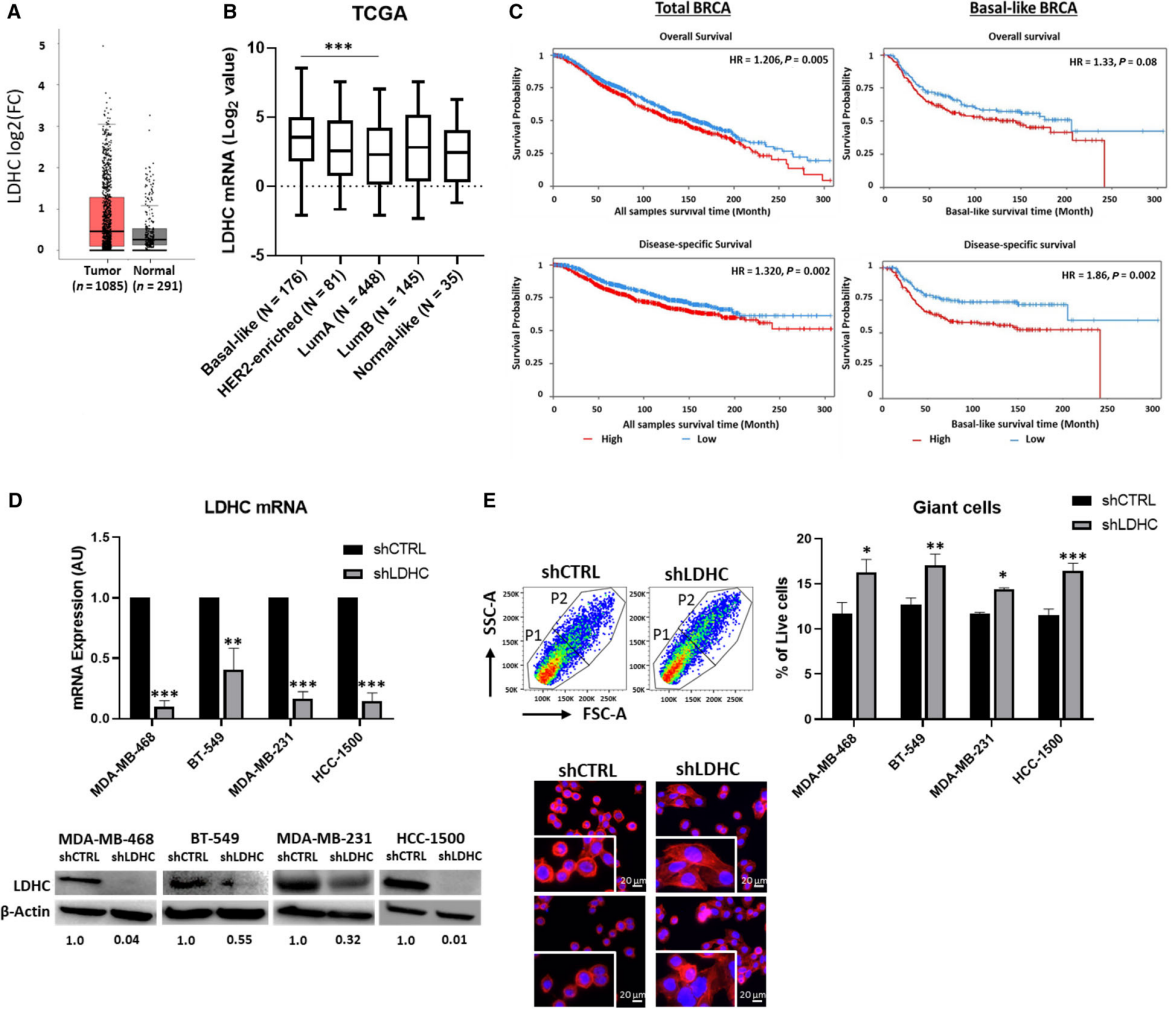
LDHC expression in breast cancer. (A) Box plot of LDHC mRNA expression in tumor and normal breast tissue using the Breast Cancer (BRCA) TCGA dataset. Differential expression analyzed with one‐way ANOVA. (B) Box plot depicting LDHC mRNA expression in intrinsic breast cancer subtypes in TCGA dataset. Statistical analysis performed using ANOVA and Tukey *post hoc* test, *** basal‐like vs luminal A. (C) Kaplan–Meier curves for overall and disease‐specific survival, stratified by median LDHC mRNA expression in the METABRIC cohort. The log rank test was used to assess survival differences. Hazards ratio (HR) and *P* values are indicated. (D) LDHC mRNA, normalized to RPLPO, and LDHC protein expression in breast cancer cell lines stably transfected with shCTRL or shLDHC expression vectors. β‐actin protein expression indicated as western blot loading control. Numbers under each lane represent mean densitometry values (arbitrary units) of LDHC signal normalized to β‐actin from three independent experiments. (E) (Top) Flow cytometry‐based quantification of giant cells. Left—representative forward scatter (FSC‐A) vs side scatter (SSC‐A) flow cytometry plots for MDA‐MB‐468 cells with cell populations sub‐grouped as P1 and P2 (giant cells). Right—frequency of giant cells (P2) in *LDHC*‐silenced compared to control cells. (Bottom) representative immunofluorescence microscopy images of MDA‐MB‐468 with nuclear (DAPI, blue) and F‐actin (red) staining (20x magnification), inserts at 2× zoom. Statistical analysis comparing shCTRL vs shLDHC performed using Student’s *t*‐test. Error bars represent standard error of mean (± SEM) from three independent replicates. *P ≤ 0.05, **P ≤ 0.01, ***P ≤ 0.001.

To confirm the testis‐specific expression of LDHC, we interrogated publicly available databases and performed in‐house qPCR for a wide range of normal tissue types. We verified that LDHC mRNA and protein expression is restricted to testis tissues with lack of or very low expression in other normal tissues (Fig. S1). Analysis of the TCGA breast cancer dataset revealed a trend of increased *LDHC* expression in breast tumor tissue compared to normal tissue (Fig. 1A). The variability in expression levels prompted us to investigate *LDHC* expression across intrinsic molecular breast tumor subtypes. *LDHC* expression was significantly increased in basal‐like tumors, the least favorable molecular subtype, compared to luminal A tumors, the most favorable subtype (Fig. 1B). Furthermore, high expression of *LDHC*, in particular in basal‐like breast tumors, was associated with adverse overall (HR = 1.33, *P* value = 0.08) and disease‐specific survival (HR = 1.86, *P* value = 0.002) (Fig. 1C). These findings are in accordance with a pro‐tumorigenic role for LDHC in less favorable subtypes such as basal‐like tumors.

### 3.2 *LDHC* silencing induces the formation of giant cancer cells

In order to investigate the role of LDHC in breast cancer, its expression was stably silenced in three basal‐like breast cancer cell lines (MDA‐MB‐468, BT‐549, MDA‐MB‐231) alongside one nonbasal, luminal A breast cancer cell line (HCC‐1500). LDHC mRNA and protein expression was significantly reduced in all cell lines using LDHC‐specific shRNA, albeit at different efficiencies, with the highest knockdown of *LDHC* in MDA‐MB‐468 and HCC‐1500 and to a lesser extent in MDA‐MB‐231 and BT‐549 (Fig. 1D). Of note, no significant changes in LDHA and LDHB were observed upon *LDHC* silencing (Fig. S2A). Silencing of *LDHC* significantly increased the number of giant cells and induced changes in the actin cytoskeleton whereby the ring‐like distribution of filamentous actin (F‐actin) stress fibers in shCTRL cells was replaced by a network of elongated actin filaments in a proportion of shLDHC cells (Fig. 1E) [30].

### 3.3 *LDHC* silencing induces genomic instability and mitotic catastrophe

**Fig. 2:**
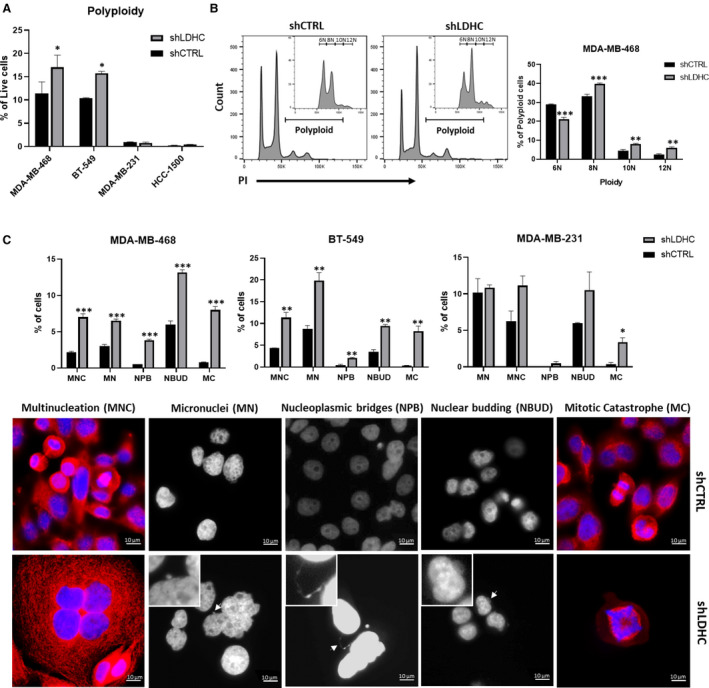
Silencing LDHC exacerbates polyploidy and nuclear aberrations. (A) Frequency of cells exhibiting polyploidy (≥ 4N ploidy) in shLDHC vs shCTRL cells, as determined by PI flow cytometry analysis. (B) Degree of polyploidy in shLDHC vs shCTRL MDA‐MB‐468 cells. Representative histograms with inserts representing genomic content of giant cells (P2). (C) (Top) Frequency of nuclear aberrations in shLDHC and shCTRL cells (*n* = 600). (Bottom) Representative immunofluorescence microscopy images of nuclear aberrations in MDA‐MB‐468 shCTRL and shLDHC cells with nuclear (DAPI, blue) and β‐tubulin (red) staining (100× magnification), inserts at 2.5× zoom. All statistical analysis comparing shCTRL vs shLDHC performed using Student’s *t*‐test. Error bars represent standard error of mean (± SEM) from three independent replicates. *P ≤ 0.05, **P ≤ 0.01, ***P ≤ 0.001. MC‐Mitotic catastrophe, MN‐Micronuclei, MNC‐Multinucleated cells, NBUD‐Nuclear budding, NPB‐Nucleoplasmic bridges.

Next, we investigated the presence and extent of genomic instability and nuclear aberrations as common features of giant cancer cells [31, 32]. We found that *LDHC* silencing increased the frequency and extent of polyploidy (≥ 4N) in MDA‐MB‐468 and BT‐549 cells (Fig. 2A,B) but not in breast cancer cells with inherent low levels of polyploidy (MDA‐MB‐231 and HCC‐1500). MDA‐MB‐468 shLDHC cells displayed an increase in the proportion of cells with 8N, 10N, and 12N polyploidy and decrease in 6N polyploidy (Fig. 2B). In addition, shLDHC cells displayed an increase in the presence of nuclear aberrations, including multinucleation (MNC), micronuclei (MN), nucleoplasmic bridges (NPB), and nuclear budding (NBUD) (Fig. 2C). As a countermeasure for increased genomic instability, cells can undergo mitotic catastrophe (MC), driving cells into an antiproliferative fate [33]. In line with this protective mechanism, a significant proportion of shLDHC cells exhibit mitotic catastrophe, featuring multiple spindle assemblies and defects in spindle formation leading to lagging chromosomes and improper mitotic segregation (Fig. 2C). We were not able to validate these observations in the HCC‐1500 cell line model due to the relatively small‐cell size impeding accurate microscopic assessment.

### 3.4 *LDHC* silencing triggers excessive DNA damage and microtubule destabilization

**Fig. 3:**
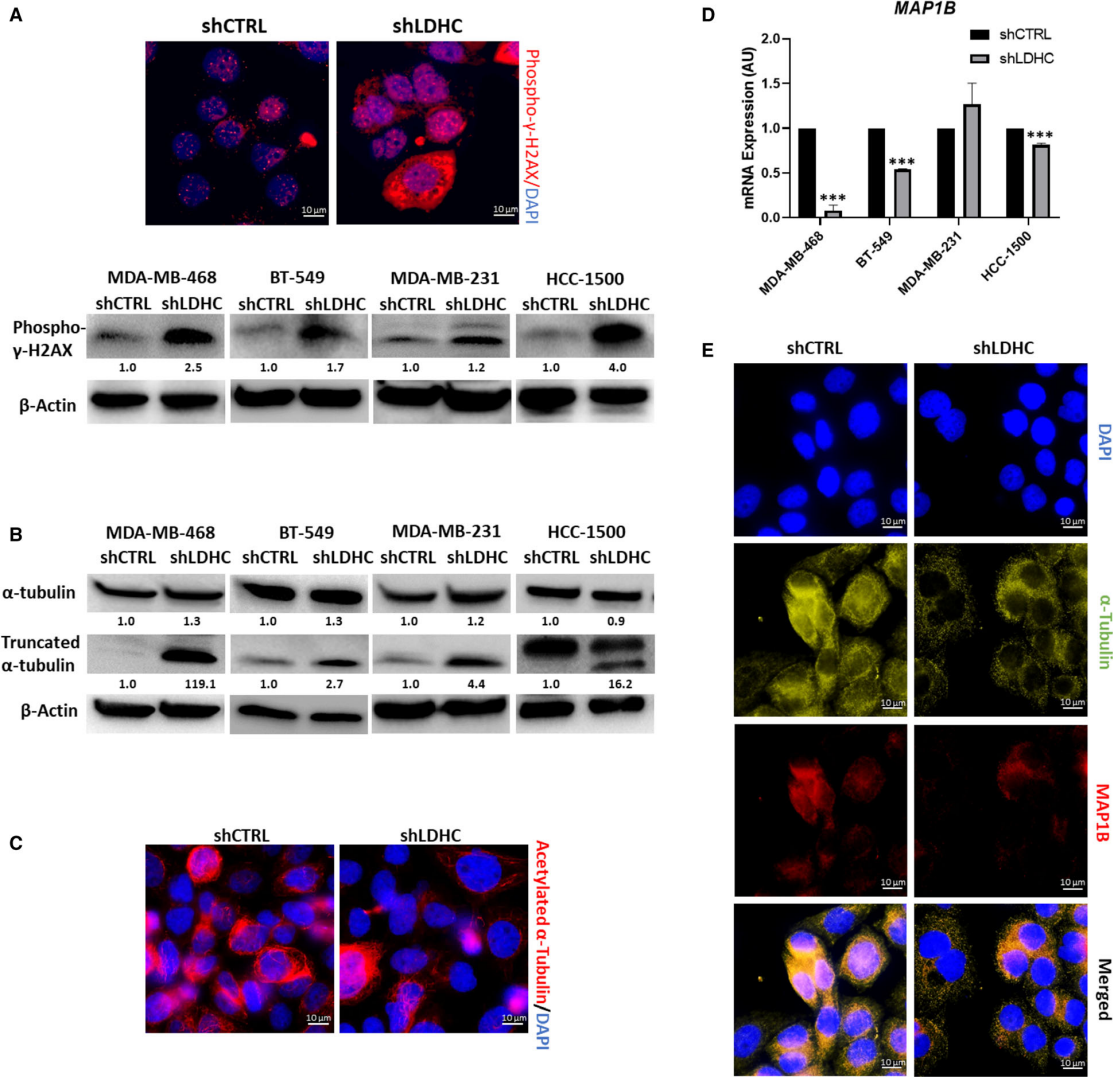
LDHC regulates DNA damage accumulation and microtubule network stability. (A) (Top) Representative immunofluorescence microscopy images of phospho‐γ‐H2AX (red) and DAPI (blue)‐stained nuclei in MDA‐MB‐468 cells (100× magnification). (Bottom) Western blot of phospho‐γ‐H2AX and β‐actin as loading control. (B) Western blot of full length and degraded α‐tubulin expression. Note that HCC‐1500 cells show an additional degraded product of α‐tubulin with slightly higher molecular weight (not quantified). (C) Representative immunofluorescence microscopy images of acetylated α‐tubulin (red) and cell nuclei (DAPI, blue) in MDA‐MB‐468 cells (100× magnification). (D) MAP1B mRNA expression, normalized to RPLPO expression. (E) Representative immunofluorescence microscopy images of α‐tubulin (green), MAP1B (red), and DAPI (blue)‐stained nuclei in MDA‐MB‐468 cells (100× magnification). For panels A and B western blots, numbers under each lane represent mean densitometry values (arbitrary units) for respective protein signal normalized to β‐actin from three independent experiments. Statistical analysis comparing shCTRL vs shLDHC performed using Student’s *t*‐test. Error bars represent standard error of mean (±SEM) from three independent replicates. ***P ≤ 0.001.

In line with an increase in mitotic catastrophe, *LDHC* silencing markedly increased the expression of phospho‐gamma‐H2AX (γ‐H2AX) in all four cell line models, indicating the presence of excess DNA damage (Fig. 3A). Assessment of DNA damage sensors upstream of the DNA damage response (DDR) pathway demonstrated that expression of ATM, ATR, DNA‐PKC, and phospho‐BRCA1 were downregulated by *LDHC* silencing, albeit to slightly different extents based on the cell line (Fig. S2B). Furthermore, *LDHC* silencing disrupted mitotic spindle organization, a common feature of cells undergoing mitotic catastrophe. More specifically, we found an increase in α‐tubulin degradation (Fig. 3B), a decrease in its acetylation of lysine residue 40 (Fig. 3C), and a more punctate staining in shLDHC cells (Fig. 3E), collectively suggesting an increase in microtubule destabilization [34, 35, 36]. In addition, the expression of microtubule‐associated protein 1B (MAP1B), involved in maintaining microtubule stability [37], was significantly downregulated by *LDHC* silencing in three out of four cell lines (Fig. 3D,E). The lack of a decrease in *MAP1B* in MDA‐MB‐231 shLDHC cells suggests that alternative MAPs may be involved in driving microtubule destabilization in these cells. Together, these findings indicate that *LDHC* silencing promotes genomic instability and mitotic catastrophe concomitant with excessive DNA damage and mitotic spindle destabilization.

### 3.5 Mitotic catastrophe‐associated cell fates

The activation of mitotic catastrophe can drive cells toward either of two cell fates: mitotic cell death or mitotic slippage. Hence, we assessed whether *LDHC* silencing facilitates either cell fate and determined the expression of key players of mitotic entry and exit.

#### 3.5.1 Increased cell death

**Fig. 4:**
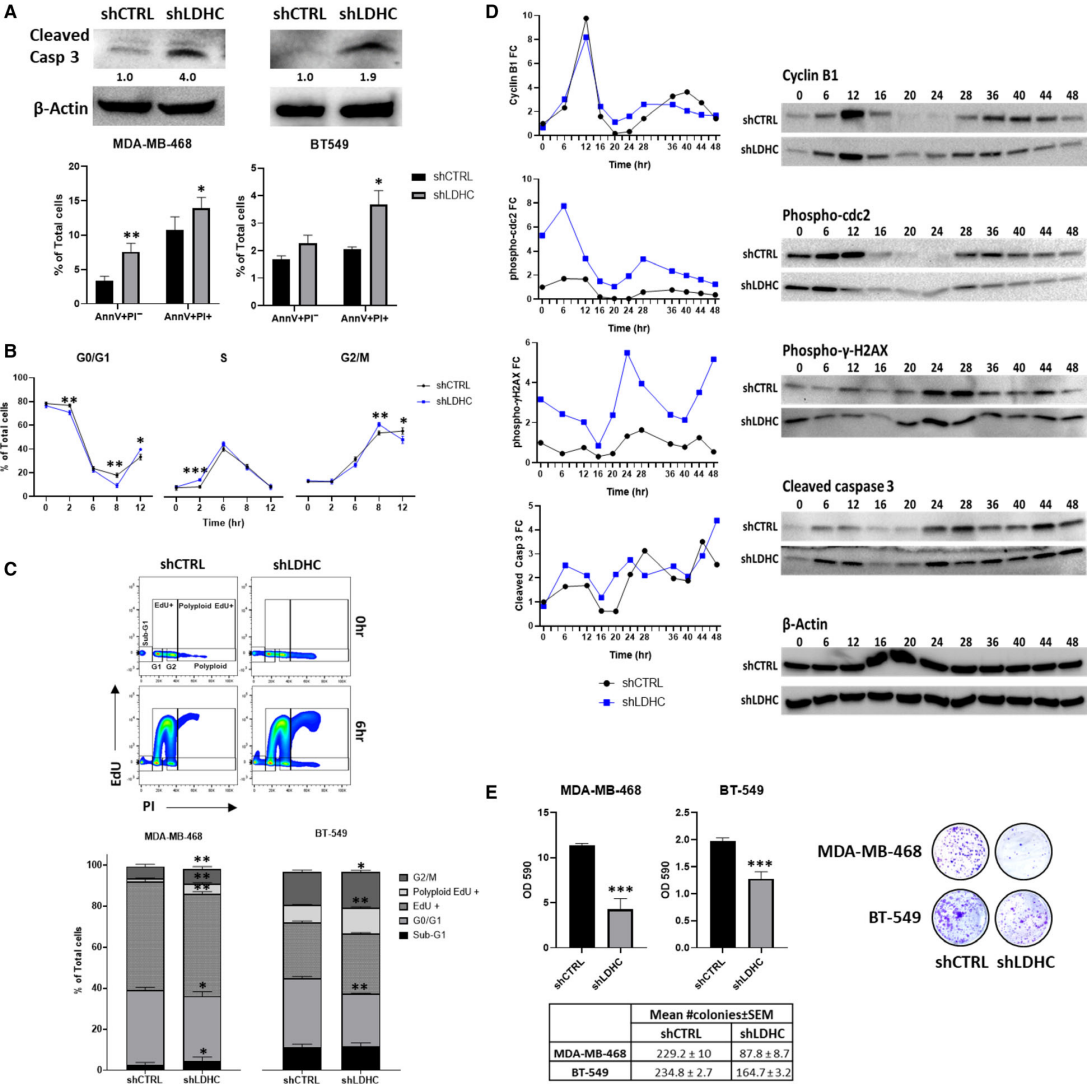
LDHC abrogation induces apoptosis, mitotic dysregulation, and decreases long‐term survival. (A) (Top) Western blot of cleaved caspase 3 expression with β‐actin as loading control. Numbers under each lane represent mean densitometry values (arbitrary units) for cleaved caspase 3 signal normalized to β‐actin from three independent experiments. (Bottom) Quantification of apoptotic cells by Annexin V/PI flow cytometry with early apoptosis defined as AnnV+PI‐ cells and late apoptosis as AnnV+PI+ cells. (B) Time course of cell cycle distribution of synchronized MDA‐MB‐468 cells using PI flow cytometry (error bars represent ± standard deviation). (C) (Top) Representative cell cycle profile of asynchronous cell populations, as determined by EdU/PI flow cytometry. (Bottom) Proportion of asynchronous MDA‐MB‐468 and BT‐549 cells in each cell cycle phase. (D) Line chart representing the fold change (FC) in protein expression over time, as determined by western blot of cyclin B1, phopho‐cdc2, phosph‐γ‐H2AX, and cleaved caspase 3 expression with representative β‐actin as loading control. Mean densitometry values (arbitrary units) of respective protein signals from three independent experiments were normalized to β‐actin, and the normalized values were used to calculate the fold change in comparison to shCTRL at 0 h. (E) (Top left) Mean OD590 for crystal violet quantification, (Bottom left) mean number of colonies, and (Right) representative image as determined by clonogenic assay. All statistical analysis comparing shCTRL vs shLDHC performed using Student’s *t*‐test. Error bars (in A, C, E) represent standard error of mean (± SEM) from three independent replicates. *P ≤ 0.05, **P ≤ 0.01, ***P ≤ 0.001.

*LDHC* silencing‐induced apoptosis in all four breast cancer cell lines, as demonstrated by an increase in caspase 3 cleavage (Fig. 4A, Fig. S2C). Furthermore, flow cytometric analysis revealed an increase in the proportion of early (Annexin V positive, PI negative) and/or late (Annexin V positive, PI positive) apoptotic cells after *LDHC* silencing (Fig. 4A, Fig. S2D,E).

#### 3.5.2 Aberrant mitosis and loss of survival

Next, we investigated whether *LDHC* silencing is associated with mitotic slippage whereby cells prematurely exit mitosis by analyzing the cell cycle distribution of synchronized cells for up to 12 h (Fig. 4B, Fig. S3A,B). In comparison with MDA‐MB‐468 shCTRL cells, shLDHC cells demonstrated rapid transition from G0/G1 to S and G2/M phase (2 and 8 h, respectively), and mitotic slippage with premature mitotic exit from the G2/M phase into the next cell cycle (12 h) (Fig. 4B). In addition, we found an increase in the proportion of shLDHC cells likely undergoing mitotic cell death (34% vs 21% sub‐G1 shCTRL at 12 h) (Fig. S3C). Similarly, analysis of BT‐549 shLDHC cells revealed rapid transition from G0/G1 to S phase (Fig. S3B) and increased cell death (12 h) (Fig. S3C). Of note, mitotic slippage in BT‐549 shLDHC cells was preceded by G0/G1 arrest in a likely attempt to repair DNA damage, and the cells displayed a significantly higher number of polyploid cells (12 h), suggesting sustained defective mitosis (Fig. S3B). Both cell line models support the hypothesis that *LDHC* silencing induces mitotic dysregulation and subsequent apoptosis. Moreover, analysis of asynchronous populations of MDA‐MB‐468 and BT‐549 cells (Fig. 4C) provided further evidence for shLDHC‐associated mitotic slippage with an increase in actively replicating polyploid cells (polyploid Edu+). Additional analyses of all four cell line models (Fig. 4C, Fig. S4A) revealed a shLDHC‐associated shift in the proportion of cells in G0/G1 (2N, EdU‐negative) and G2/M (4N, EdU‐negative) with MDA‐MB‐468 and HCC‐1500 shLDHC cells also showing an increase in sub‐G1 apoptotic cells. Together, these results suggest that silencing *LDHC* is associated with both mitotic slippage and arrest, followed by cell death.

To gain insight into the molecular mechanisms driving progression through mitosis, we assessed the activation status of the cyclin B1‐cdc2 complex in the MDA‐MB‐468 cell line that demonstrated high *LDHC* silencing efficiency and a robust phenotype (Fig. 4D). Analysis of the expression of cyclin B1 and of inactive phosphorylated cdc2 (Tyr15 phospho‐cdc2) revealed remarkable differences in expression dynamics in shLDHC cells compared to shCTRL cells over a time period of 48 h (approximately two cycles of mitotic entry). Both control and *LDHC*‐silenced cells displayed the characteristic premitotic peak in cyclin B1 expression at 12‐h postsynchronization, and however, shLDHC cells showed an earlier decline in phospho‐cdc2 (6–16 vs 12–16 h in shCTRL) in accordance with a more rapid G2/M transition and initiation of mitosis. Subsequently, shLDHC cells displayed an earlier second peak in cyclin B1 expression followed by cdc2 dephosphorylation (28 h vs 40 h in shCTRL), indicating that shLDHC cells prematurely entered the second round of mitosis. Of note, the earlier peak of cyclin B1 and phospho‐cdc2 expression in shLDHC cells are followed by a slower rate of degradation. Moreover, their expression does not decline to baseline levels after the first round of mitosis (20–24 h). In concordance with our cell cycle profiling data, these observations suggest that *LDHC* silencing may result in two cell subpopulations, whereby one subset of cells undergoes mitotic slippage and the other experiences prolonged premitotic arrest with likely subsequent cell death as a result of excessive, unrepaired DNA damage [38, 39]. Indeed, it is well known that the balance of two opposing networks, involving cyclin B1 degradation and caspase cleavage, determine mitotic cell fate [40]. Analysis of phospho‐γ‐H2AX levels demonstrated a high level of DNA damage in shLDHC cells at 0 h and at the mitotic phases (24 and 48 h) (Fig. 4D). Furthermore, assessment of caspase 3 cleavage (Fig. 4D) provides evidence of a sustained increase in shLDHC cell death throughout the cell cycle (6–12 and 20 h onwards) and in subsequent cell cycles (48 h).

In addition to undergoing apoptosis, mitotic catastrophe can also drive cells to enter a state of senescence as observed in HCC‐1500 shLDHC cells but not in the other three cell lines (Fig. S4B). Strikingly, silencing of *LDHC* significantly decreased the colony formation ability, hence long‐term survival, of all four breast cancer cell lines (Fig. 4E, Fig. S4C).

### 3.6 *LDHC* silencing dysregulates multiple cell cycle checkpoints

**Fig. 5:**
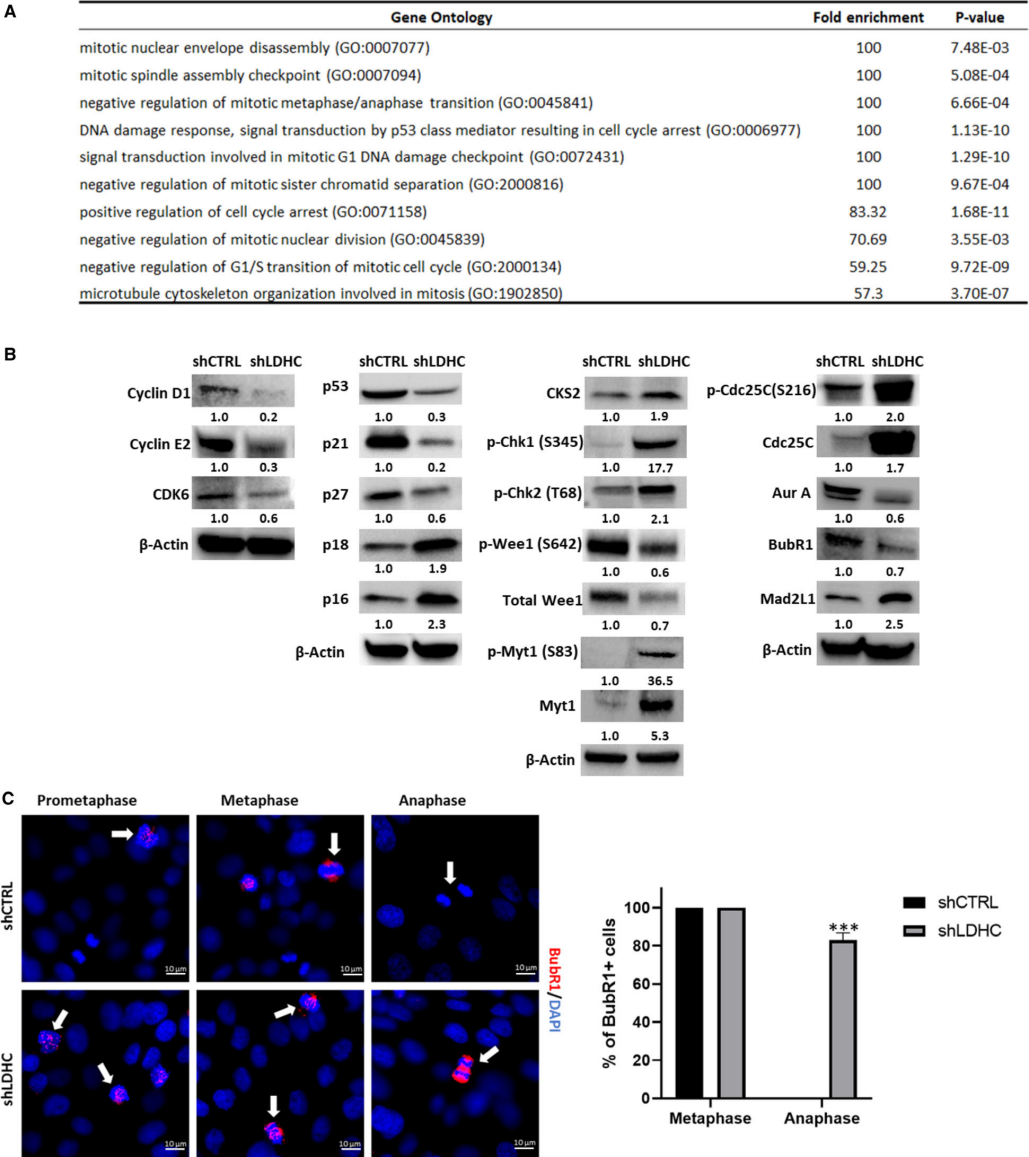
LDHC and cell cycle regulation of MDA‐MB‐468 cells. (A) GO enrichment of differentially expressed genes after *LDHC* silencing as determined by the Cell Cycle RT2 Profiler qPCR array. (B) Western blot of cell cycle regulator protein expression with β‐actin as loading control. Numbers under each lane represent mean densitometry values (arbitrary units) for respective protein signal normalized to β‐actin from three independent experiments. (C) Representative immunofluorescence microscopy images of BubR1 (red) and DAPI‐stained nuclei (blue) in prometaphase, metaphase, and anaphase stages of mitosis (indicated by white arrows, 100× magnification). All statistical analysis comparing shCTRL vs shLDHC performed using Student’s t‐test. Error bars represent standard error of mean (± SEM) from three independent replicates with *n* = 90 cells for each condition. ***P ≤ 0.001.

Based on our observations of increased mitotic dysregulation by *LDHC* silencing, we explored potential defects in G1/S, intra‐S, G2/M, and Spindle Assembly checkpoint (SAC) regulators using the MDA‐MB‐468 cell line model. Differential gene expression and gene ontology analysis of 84 cell cycle‐related genes revealed an enrichment of genes involved in dysregulated DNA damage response and impaired mitotic fidelity in *LDHC*‐silenced cells (Fig. 5A, Table S2).

Furthermore, we found that *LDHC* silencing altered the protein expression of cell cycle regulators at multiple checkpoints. For instance, the expression of the G1/S checkpoint regulators cyclin D1, cyclin E2, and cyclin‐dependent kinase (CDK)‐6 was downregulated in shLDHC cells (Fig. 5B), suggesting that less cells reside in the G1 phase or that the G1 phase is shortened, which is in line with our cell cycle profile observations (Fig. 4C). The observed upregulation of INK4 type CDK inhibitors (p16, p18) and associated downregulation of p53 and its downstream targets p27 and p21 [41] further supports the likelihood of G1/S checkpoint dysregulation in shLDHC cells. In addition, *LDHC* silencing increased the expression of cyclin‑dependent kinase subunit 2 (CKS2), likely mediating an override of the intra‐S phase checkpoint in the presence of replication stress [42] and facilitating transition from G2 to M phase [43] (Fig. 5B, Table S2). Expression analysis of the negative G2/M checkpoint regulators Wee1 and Myt1 demonstrated a decrease in total and active phosphorylated Wee1 (Ser642), and conversely an increase in total and inactive phosphorylated Myt1 (Ser83) in shLDHC cells, supporting our previous observations that *LDHC* silencing promotes mitotic entry. On the other hand, we found an increase in phosphorylated checkpoint proteins Chk1 (Ser345) and Chk2 (Thr68), indicating the presence of single‐ and double‐strand DNA breaks, that in turn inactivate Cdc25C by phosphorylation at Ser216, resulting in G2/M arrest. Collectively, these findings corroborate the presence of two shLDHC cell subpopulations; one population that undergoes G2/M arrest and another that undergoes checkpoint adaptation and slippage [44]. Further evidence for the existence of two shLDHC cell populations was provided by the assessment of mitotic/SAC regulators such as Aurora A [45, 46] and Mad2L1. We found that total and phosphorylated Aurora A expression were significantly reduced in shLDHC cells (Fig. 5B, Table S2) and as such may result in the loss of an active SAC with premature transition from metaphase into anaphase [47]. In addition, expression of Mad2L1 was upregulated after *LDHC* silencing, possibly triggering cells to undergo long‐term SAC activation followed by mitotic slippage [48] (Fig. 5B, Table S2). In contrast, we observed a slight decrease in the expression of the kinetochore‐associated protein BubR1 in shLDHC cells, thus impairing accurate spindle attachment and chromosome segregation. Although the cellular localization of BubR1 did not differ in the pro‐metaphase between shCTRL and shLDHC cells, its expression at the kinetochores and at segregating chromosomes in the metaphase and early anaphase was dysregulated in shLDHC cells, indicating defective sister chromatid segregation and prevention of anaphase onset (Fig. 5C). In line with this, the majority of shLDHC cells in anaphase remained BubR1 positive whereas shCTRL cells completely lost BubR1 expression going from metaphase to anaphase.

In conclusion, assessment of the expression of cell cycle regulators in the MDA‐MB‐468 cell line confirmed the presence of two cell subpopulations after *LDHC* silencing: one subpopulation undergoing mitotic arrest at the G2/M and/or SAC checkpoint and another undergoing mitotic slippage. This phenotype of mitotic dysregulation upon *LDHC* silencing was observed in all four breast cancer cell lines, and however, the molecular mechanistic underpinnings of these observations may vary between cell lines based on their genetic landscape.

### 3.7 *LDHC* silencing sensitizes cancer cells to DNA damage repair inhibitors and DNA damage inducers

**Fig. 6:**
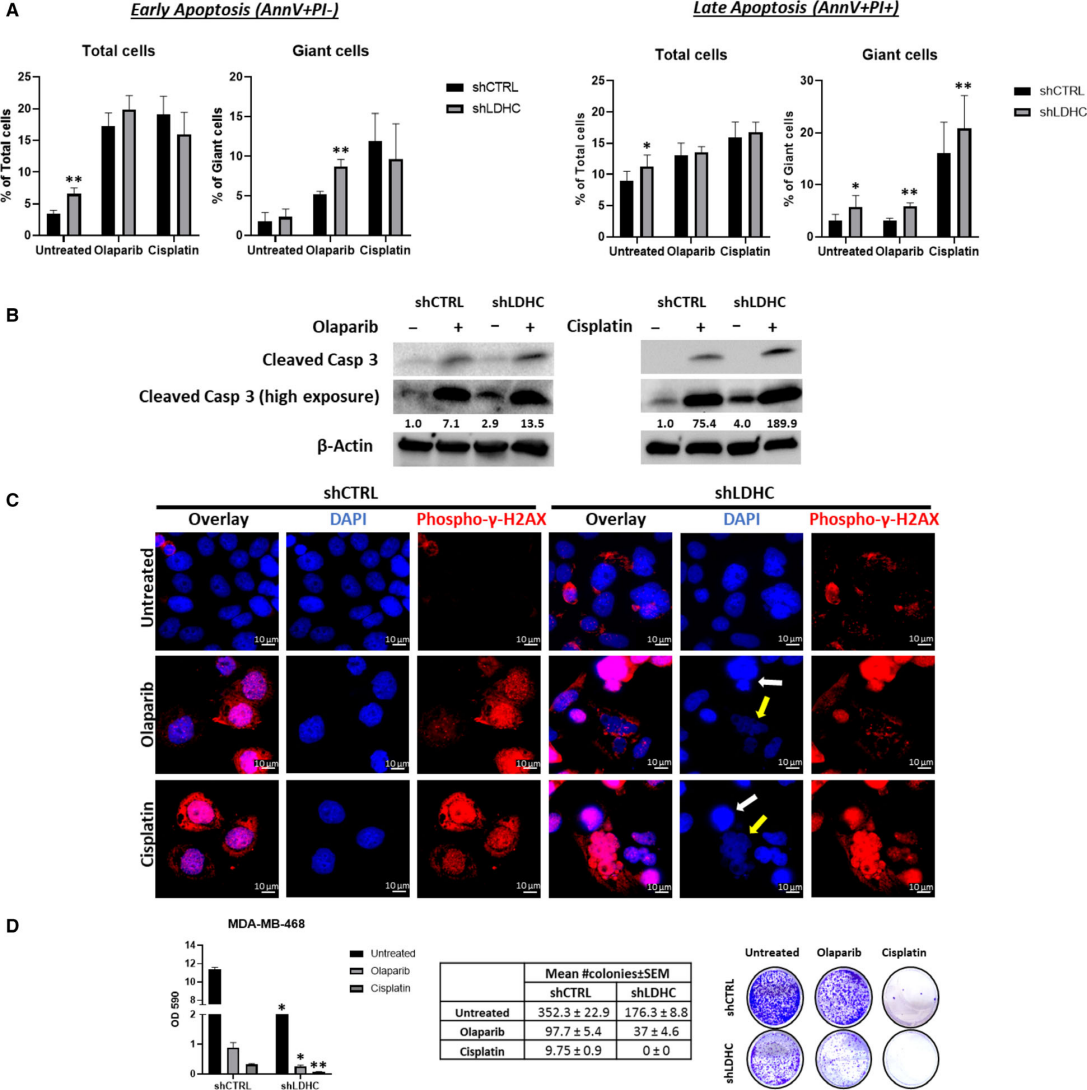
*LDHC* silencing improves sensitivity of MDA‐MB‐468 cells to DNA damage inducers and DNA damage repair inhibitors. (A) Annexin V/PI flow cytometric quantification of apoptosis after 72 h of treatment with olaparib or cisplatin. (B) Western blot of cleaved caspase 3 protein expression with β‐actin as loading control. Numbers under each lane represent mean densitometry values (arbitrary units) for cleaved caspase 3 signal (high exposure blot) from three independent experiments normalized to β‐actin. (C) Representative immunofluorescence microscopy images of phospho‐γ‐H2AX (red) and DAPI‐stained nuclei (100× magnification). Yellow arrows indicate early apoptotic cells, and white arrows indicate late apoptotic cells. (D) (Left) Mean OD590 for crystal violet quantification, (Middle) mean number of colonies, and (Right) representative images taken at 14 days of culture post‐treatment (72 h). All statistical analysis comparing shCTRL vs shLDHC performed using Student’s *t*‐test. Error bars represent standard error of mean (± SEM) from three independent replicates. *P ≤ 0.05, **P ≤ 0.01.

DNA damage repair inhibitors and DNA damage‐inducing agents, including the Poly ADP‐ribose polymerase (PARP)‐inhibitor olaparib and cisplatin, are widely used anticancer drugs with particular benefit to patients with defects in DDR pathways such as BRCA1/2‐positive or ‘BRCAness’ basal breast cancer patients [49, 50]. Since we demonstrated that *LDHC* silencing increases DNA damage accumulation in breast cancer cells with alterations in the expression of DNA damage sensors upstream of the DDR pathway, we explored the sensitivity of shLDHC cells to olaparib and cisplatin. Treatment with either drug further induced apoptosis in giant shLDHC cells compared to shCTRL cells (Fig. 6A, Fig. S5A–C). In accordance, olaparib and cisplatin treatment augmented the expression of cleaved caspase 3 in shLDHC cells (Fig. 6B). Moreover, we observed a significant increase in DNA damage after olaparib and cisplatin treatment, albeit at similar levels in both shCTRL‐ and shLDHC‐treated cells (Fig. 6C). However, shLDHC cells with excessive DNA damage displayed apoptotic nuclear features such as highly condensed DNA and nuclear fragmentation. Finally, the colony formation ability of shLDHC cells was further compromised in a cell‐line‐dependent manner, by about twofold after treatment with olaparib and greater than fourfold after treatment with cisplatin, compared to shCTRL treated cells (Fig. 6D, Fig. S5D). The MDA‐MB‐468 and BT‐549 shLDHC cells were the most sensitive and the MDA‐MB‐231 shLDHC cells were the least sensitive to treatment.

## 4 Discussion

**Fig. 7:**
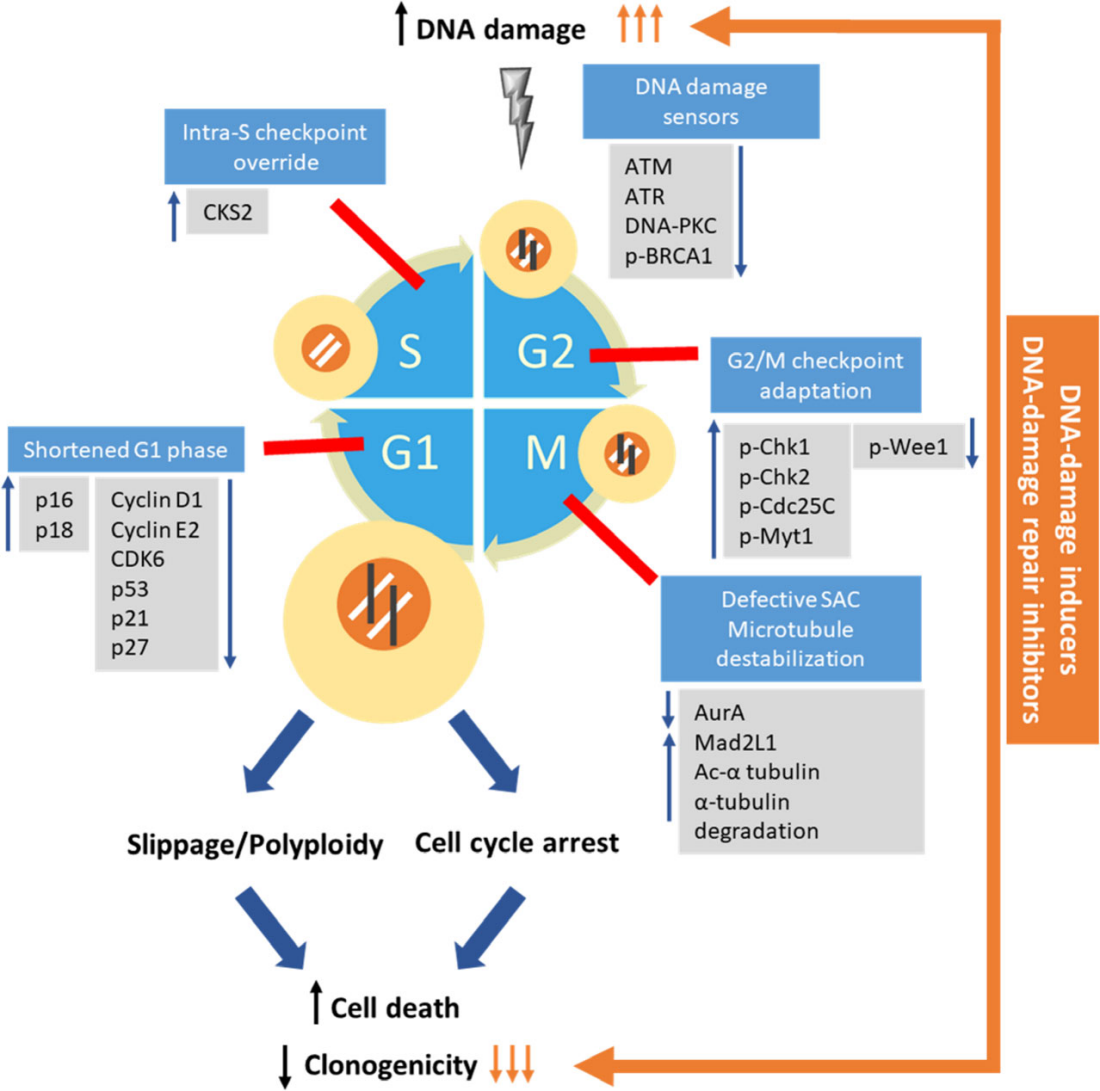
Schematic model of the effect of LDHC targeting on cell cycle checkpoints and cell fate. Silencing of *LDHC* in breast cancer cells increases the rate of mitotic entry by dysregulation of several cell cycle checkpoints, resulting in a shorter cell cycle. Firstly, targeting LDHC shortens the G1 phase and downregulates several molecules controlling the G1/S checkpoint and mediates override of the intra‐S phase checkpoint. Next, G2/M checkpoint regulators are aberrantly expressed in LDHC‐silenced cells. Together, this results in excessive DNA damage (black lines) due to the decreased expression of DNA damage sensors upstream of the DDR pathway and hence lack of functional repair mechanisms. On mitotic entry, the unstable microtubule network in LDHC‐silenced cells triggers defective chromosome segregation, thus activating the spindle assembly checkpoint (SAC). While a proportion of cells with high levels of genomic instability undergo arrest and cell death, an additional population of cells with lower levels of instability undergo long‐term SAC activation and mitotic slippage in the absence of cytokinesis. These cells form giant polyploid cancer cells that subsequently undergo cell death or senescence, ultimately diminishing clonogenicity or long‐term survival of cancer cells. Additionally, targeting LDHC in combination with DNA‐damaging/DNA‐repair‐inhibiting agents (orange arrows) synergistically dysregulates the DNA damage repair pathways and promotes cell death pathways, resulting in significant loss of cell survival. The G1/S, intra‐S, G2/M, and SAC checkpoints are depicted as red lines. The molecular regulators depicted were found to be involved in LDHC‐silenced MDA‐MB‐468, and however, we cannot exclude that there may be cell‐line‐dependent differences in regulators.

Cancer testis antigens, characterized by tumor‐restricted expression and immunogenicity, are attractive candidate targets for cancer therapy. Gaining insight into their role in tumorigenesis could facilitate the identification of individual highly tumor‐specific CTAs with therapeutic potential that may synergistically improve the efficacy of available cancer therapies [51]. While cancer cells commonly exhibit deficiencies in DNA damage repair pathways, there appears to be a threshold whereby low levels of genomic instability drive tumorigenicity while excess genomic aberrations compromise cellular fitness through cell cycle arrest, senescence or cell death [31]. Here, we report that targeting of LDHC in breast cancer cells has the potential to tip the fine balance between tolerable and excessive levels of genomic damage in favor of the latter and as such to sensitize cancer cells to DNA damage inducers and DNA damage repair inhibitors (Fig. 7). Targeting the LDHA isozyme or total LDH has been previously reported to dysregulate the cell cycle, dampen the DNA damage response and induce apoptosis in numerous cancer cell lines [52, 53, 54]. Whereas it is likely that LDH family members may share pro‐oncogenic functions, LDHC is in a unique position as anticancer target since it features a restricted expression in healthy somatic tissues compared to LDHA and LDHB which are abundantly expressed in heart and liver tissues. Moreover, our findings suggest that LDHC may exert its effects on genomic integrity and cancer cell survival independent of LDHA/B as we did not observe any compensatory increase in LDHA/B expression for the loss or reduction of LDHC expression (Fig. S2A). Hence, LDHC expression in tumor cells could be targeted with minimal off‐target effects, thereby affecting long‐term tumor cell survival.

Through a comprehensive analysis of four breast cancer cell lines, we demonstrated that *LDHC* silencing induces DNA damage accumulation and impairs mitotic fidelity by dysregulating multiple cell cycle checkpoints and microtubule assembly, ultimately resulting in mitotic catastrophe, apoptosis and reduced long‐term survival (Fig. 7, Table S3). Of note, these observations were consistent in three basal‐like breast cancer cell lines and in one luminal A cell line, indicating the broad applicability of LDHC function in maintaining mitotic fidelity. Although the various cell lines share similar cell fates upon *LDHC* silencing, their genetic background varies and the specific molecular mediators and mechanisms involved may be cell line‐dependent. In conjunction with *LDHC* knockdown efficiency, differences in genetic mutational status and inherent susceptibility to genomic instability may partly explain some of the mechanistic differences observed in the various cell lines. For instance, the genetic landscapes of the cell lines differ in the mutational status of the Rb and p53 tumor suppressor genes that regulate the G1/S checkpoint (Table S3). As such, cell lines with loss of Rb and/or mutant p53 rely more heavily on a functional G2/M DNA damage checkpoint for tumor propagation, which may be reflected in molecular dysregulation nuances induced by *LDHC* silencing [55]. Furthermore, we show in p53 mutant MDA‐MB‐468 cells that *LDHC* silencing reduced the expression of the oncogenic, mutant form of p53, thus contributing to the loss‐of‐survival phenotype [56]. Conversely, HCC‐1500 cells lack p53 expression and display a more moderate phenotype upon *LDHC* silencing. This raises the question whether breast tumors with oncogenic p53 mutations that express high levels of *LDHC* (Fig. S6) could be more susceptible to LDHC targeting through disruption of the G2/M and SAC checkpoints.

Interestingly, although none of the four cell lines in our study harbor *BRCA* mutations, we observed DNA damage accumulation upon *LDHC* silencing suggesting that DNA repair pathways are likely impaired. Analysis of key molecules involved in DNA repair signaling revealed dysregulation of DNA damage sensors upstream of the DDR pathway in *LDHC*‐silenced cells. In addition, *LDHC* silencing in MDA‐MB‐468 cells, which display a greater *LDHC* knockdown efficiency and a robust phenotype, downregulated the expression of Aurora A and its downstream mediator Wee1 and inactivated Myt1. As such, *LDHC* silencing potentially induces disruption of the G2/M checkpoint, allowing cells to proceed into mitosis with excess unrepaired DNA damage [57, 58]. Moreover, Aurora A plays a critical role in microtubule nucleation and elongation indicating that its reduced expression, together with the loss of the microtubule‐associated protein MAP1B, is potentially involved in the *LDHC* silencing‐induced microtubule instability [59]. In addition to DNA damage accumulation attributed to dysregulated mitosis and DNA repair signaling, it remains to be determined if *LDHC* silencing affects the levels of DNA damage inducers such as reactive oxygen species or impairs the functionality of NAD+‐dependent enzymes such as PARPs and sirtuins, involved in maintaining genomic stability, through modulation of pyruvate‐lactate interconversion and consequently NAD+ and NADH levels [60, 61]. Additionally, future work involving live cell imaging of mitotic phenotypes and the DNA damage response in *LDHC*‐silenced cells will provide molecular insight into the temporal dynamics of genomic instability.

Given the increase in DNA damage and dysregulated expression of DNA damage sensors upon *LDHC* silencing, we speculated that, in analogy with treatment response of BRCA‐deficient tumor cells, *LDHC* silencing could improve the response to DNA damage response targeted therapy such as DNA damage inducers or DNA damage repair inhibitors that rely on DNA damage‐induced mitotic catastrophe to trigger cell death [62, 63]. Indeed, we found that silencing *LDHC* sensitizes breast cancer cells to olaparib and cisplatin albeit at different extents, thereby improving the efficacy of either treatment with subtle differences in cell line sensitivity.

Our findings indicate that targeting of LDHC likely interferes with tumor cell survival by a multitude of mechanisms affecting genomic stability, including dysregulated cell cycle progression. To date, several cell cycle inhibitors are currently under investigation or are implemented in breast cancer management, including inhibitors against Wee1 [64], Aurora A [65], CDK6 [66], mutant p53 [67], and microtubule inhibitors [68]. However, treatment with these inhibitors as single agents shows low response rates and results in the development of acquired resistance [66, 68, 69]. Of note, we found that targeting of *LDHC* negatively impacts each of these cell cycle regulators (Fig. 7), indicating that LDHC may prove to be a therapeutically superior target over individual cell cycle molecule inhibitors. Moreover, the tumor‐specific expression of LDHC allows precise targeting of cancer cells with limited off‐target effects, which is an improvement over currently available cell cycle inhibitors that affect any dividing somatic cells. Further, the combination effect of LDHC abrogation and DNA damage response targeted therapy may allow reducing the drug dosage of the latter thereby lowering the risk of toxic side effects such as myelosuppression and nephrotoxicity.

## 5 Conclusion

To conclude, we believe that developing LDHC‐specific therapeutic interventions using specific chemical inhibitors or siRNA delivery approaches presents an attractive paradigm to enhance the efficacy of current drugs and to improve clinical outcome in breast cancer. Although promising, the observations from this *in vitro* study on four breast cancer cell lines remain to be confirmed in *in vivo* models to corroborate the notion of targeting LDHC to improve clinical outcome of LDHC^high^ breast cancer patients with poor prognosis, who may benefit from treatment with DNA damage response targeted therapy using DNA damage inducers or PARPi, which is currently only prescribed to *BRCA*‐mutant patients who constitute a mere 9–15% of TNBCs [70]. Notably, noncommercial oxamic acid analogues with high affinity and potency against LDHC have been described, and however, these have not yet been investigated for their anticancer properties [71].

## Conflict of interest

The authors declare no conflict of interest.

## Author contributions

AN was responsible for the study design and methodology, performed the formal analysis and data visualization, interpreted the data and wrote the original draft. JD conceptualized and supervised the study, contributed to data interpretation, edited the draft manuscript, and acquired funding.

## Supporting information


**Fig. S1**. LDHC expression across normal tissues.

**Fig. S2**. *LDHC* silencing does not alter LDHA/LDHB expression, and increases DNA damage and apoptosis.

**Fig. S3**. Aberrant cell cycle upon *LDHC* silencing. Aberrant cell cycle upon *LDHC* silencing.

**Fig. S4**. Aberrant cell cycle and cell fate upon *LDHC* silencing.

**Fig. S5**. *LDHC* silencing improves sensitivity of breast cancer cells to DNA damage inducers and DNA damage repair inhibitors.

**Fig. S6**. *LDHC* expression in *TP53* mutant breast cancer.

**Table S1**. List of antibodies.

**Table S2**. Differentially expressed cell cycle genes.

**Table S3**. Summary of observed characteristics in *LDHC*‐silenced breast cell lines.

## Data Availability

The raw data that support the findings of this study are available from the corresponding author [juliedecock80@gmail.com] upon reasonable request.
