# Supplementary figures and images for: Targeting of lactate dehydrogenase C dysregulates the cell cycle and sensitizes breast cancer cells to DNA damage response targeted therapy
Source: Mol Oncol. 2021 Jun 13;16(4):885–903. doi: 10.1002/1878-0261.13024 (PMC8847988; doi:10.1002/1878-0261.13024)

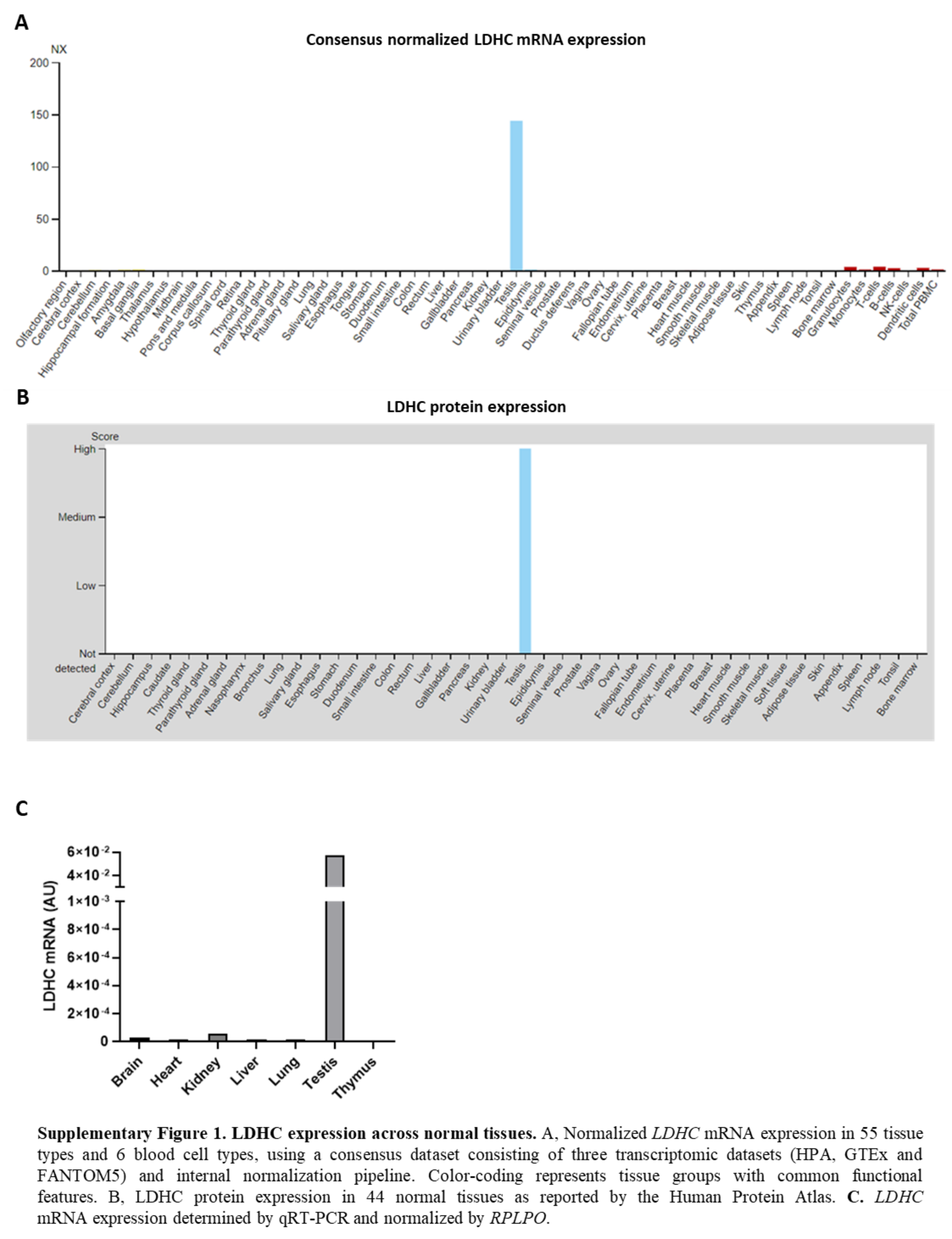

Supplement: Supplementary file 1 — Fig. S1. LDHC expression across normal tissues. [file MOL2-16-885-s007.png]

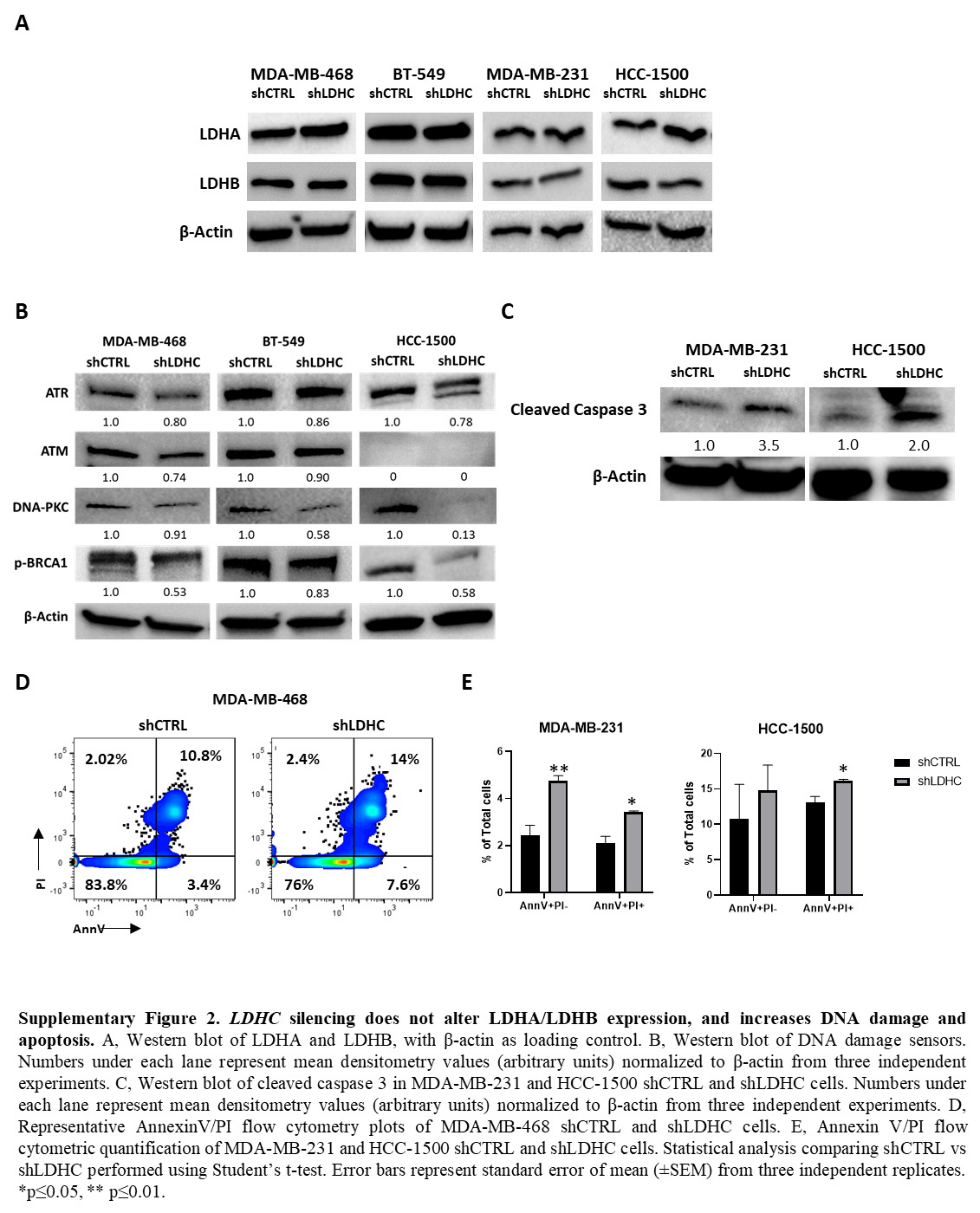

Supplement: Supplementary file 2 — Fig. S2. LDHC silencing does not alter LDHA/LDHB expression, and increases DNA damage and apoptosis. [file MOL2-16-885-s002.png]

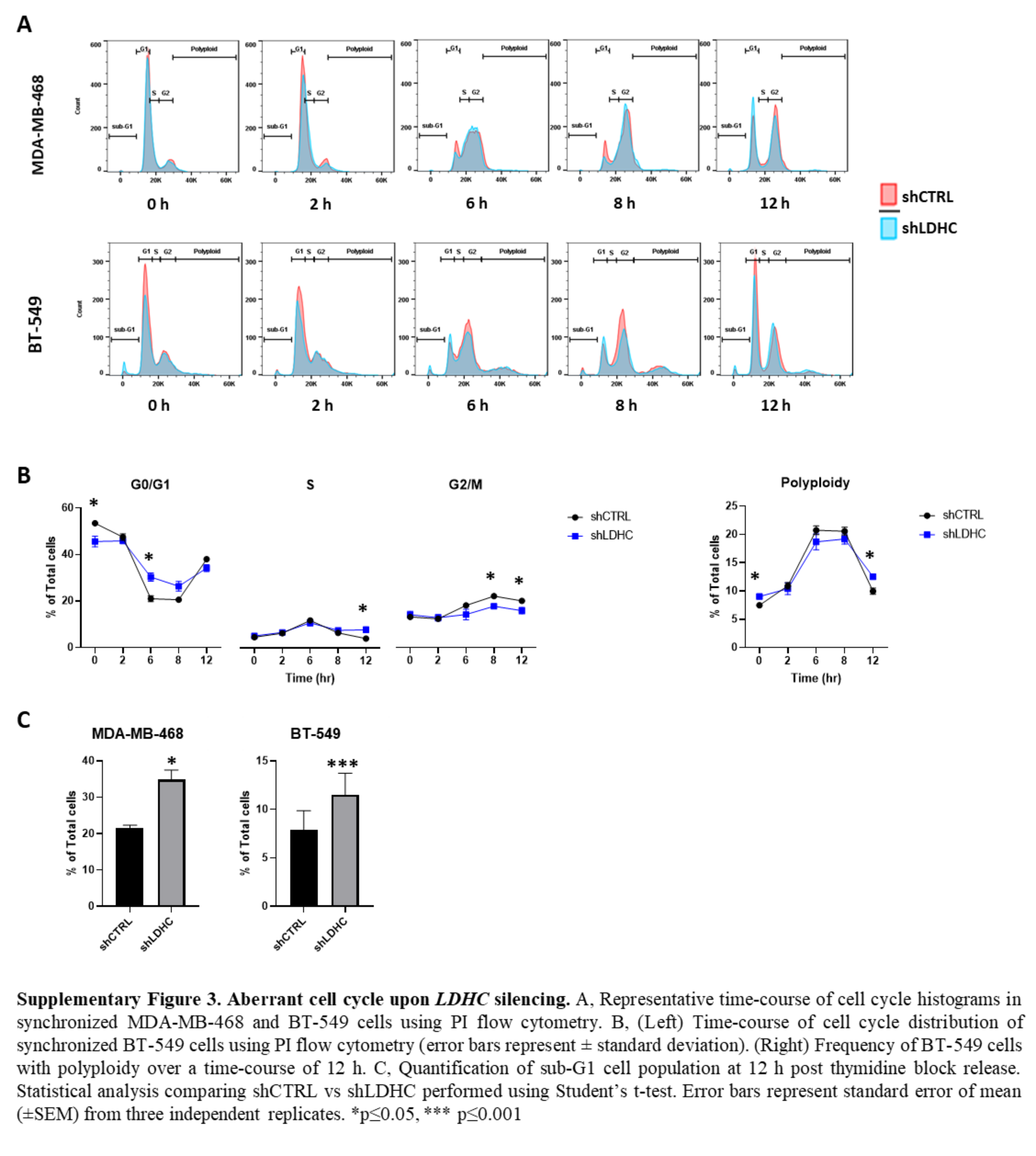

Supplement: Supplementary file 3 — Fig. S3. Aberrant cell cycle upon LDHC silencing. Aberrant cell cycle upon LDHC silencing. [file MOL2-16-885-s006.png]

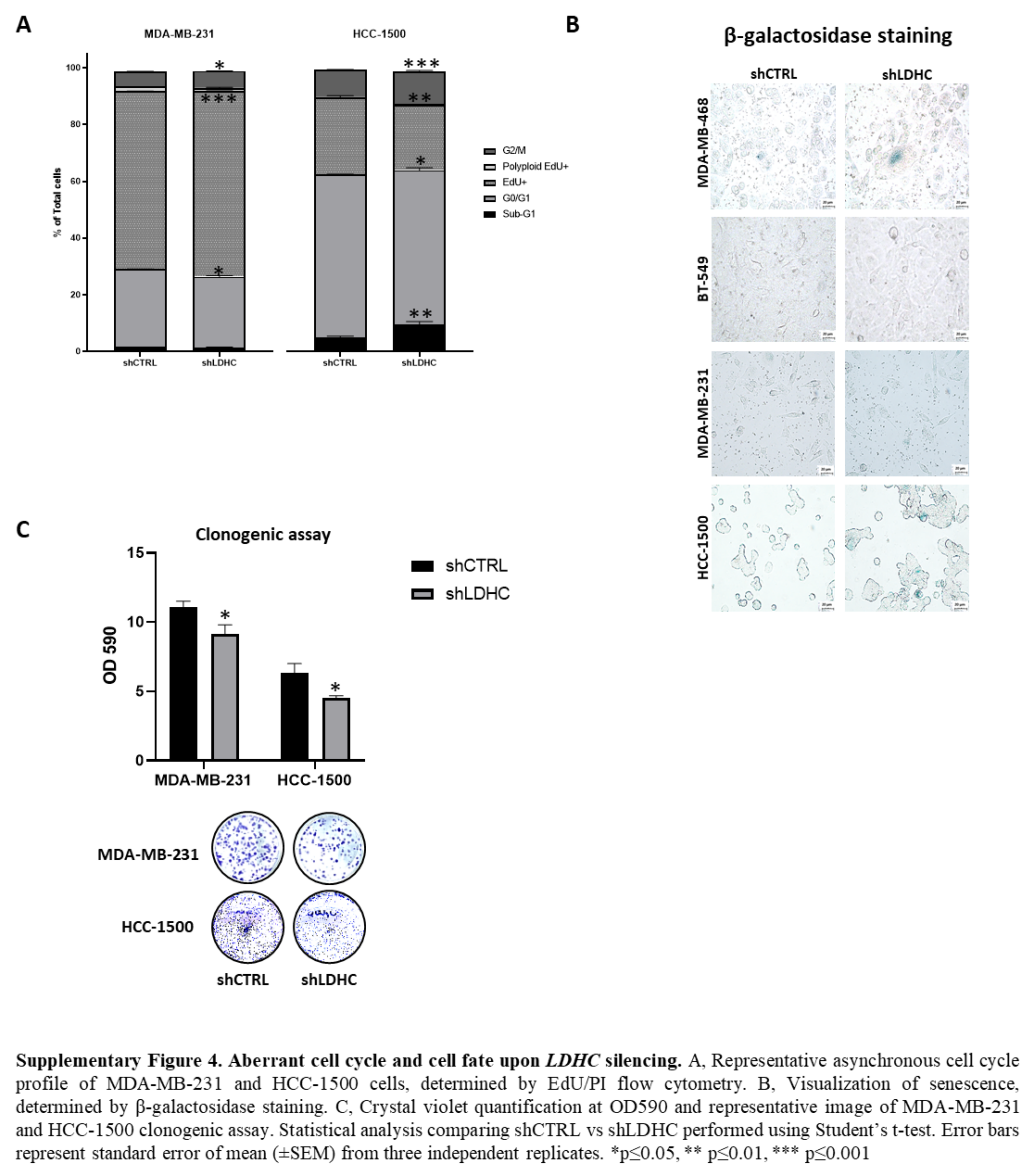

Supplement: Supplementary file 4 — Fig. S4. Aberrant cell cycle and cell fate upon LDHC silencing. [file MOL2-16-885-s004.png]

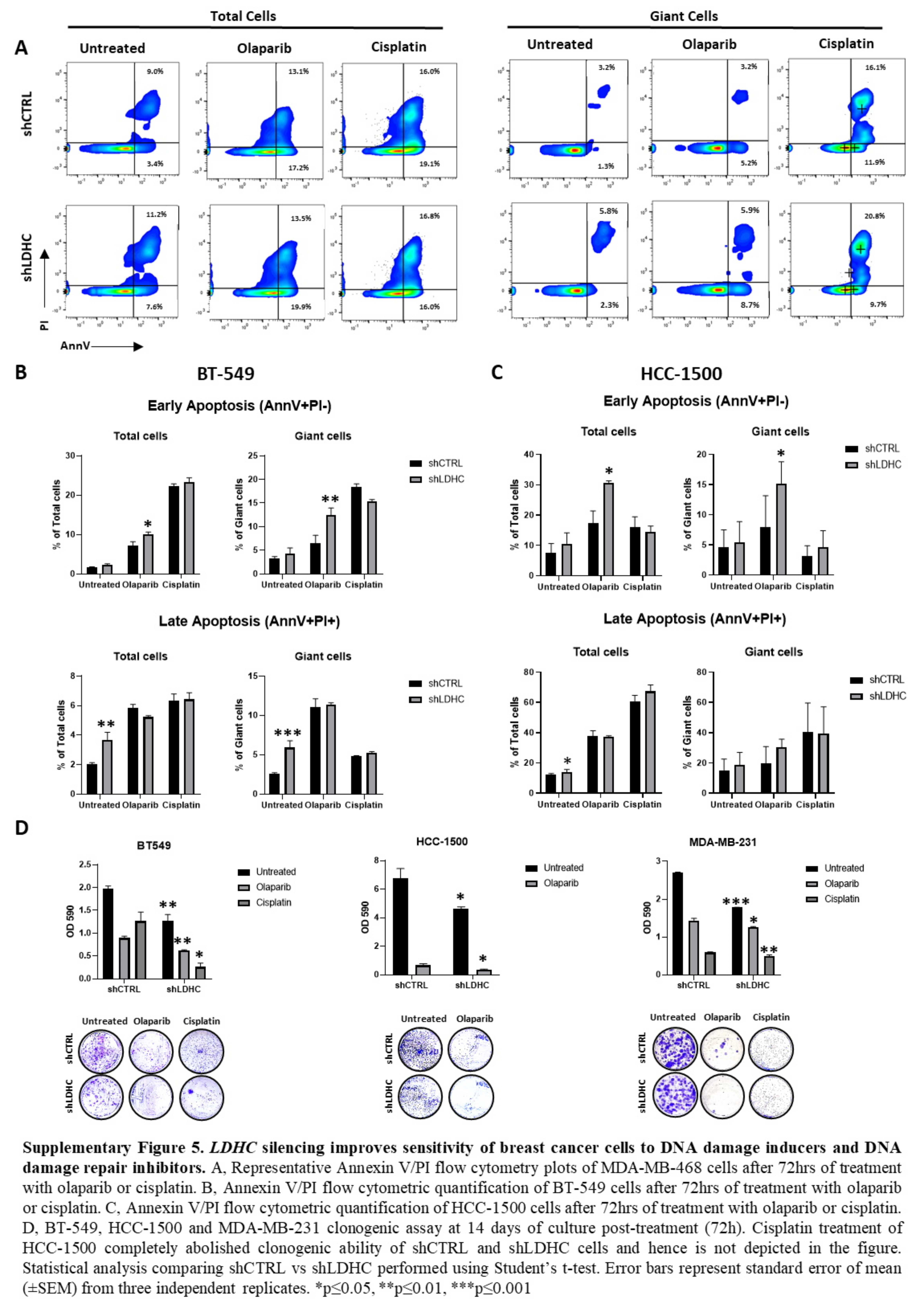

Supplement: Supplementary file 5 — Fig. S5. LDHC silencing improves sensitivity of breast cancer cells to DNA damage inducers and DNA damage repair inhibitors. [file MOL2-16-885-s008.png]

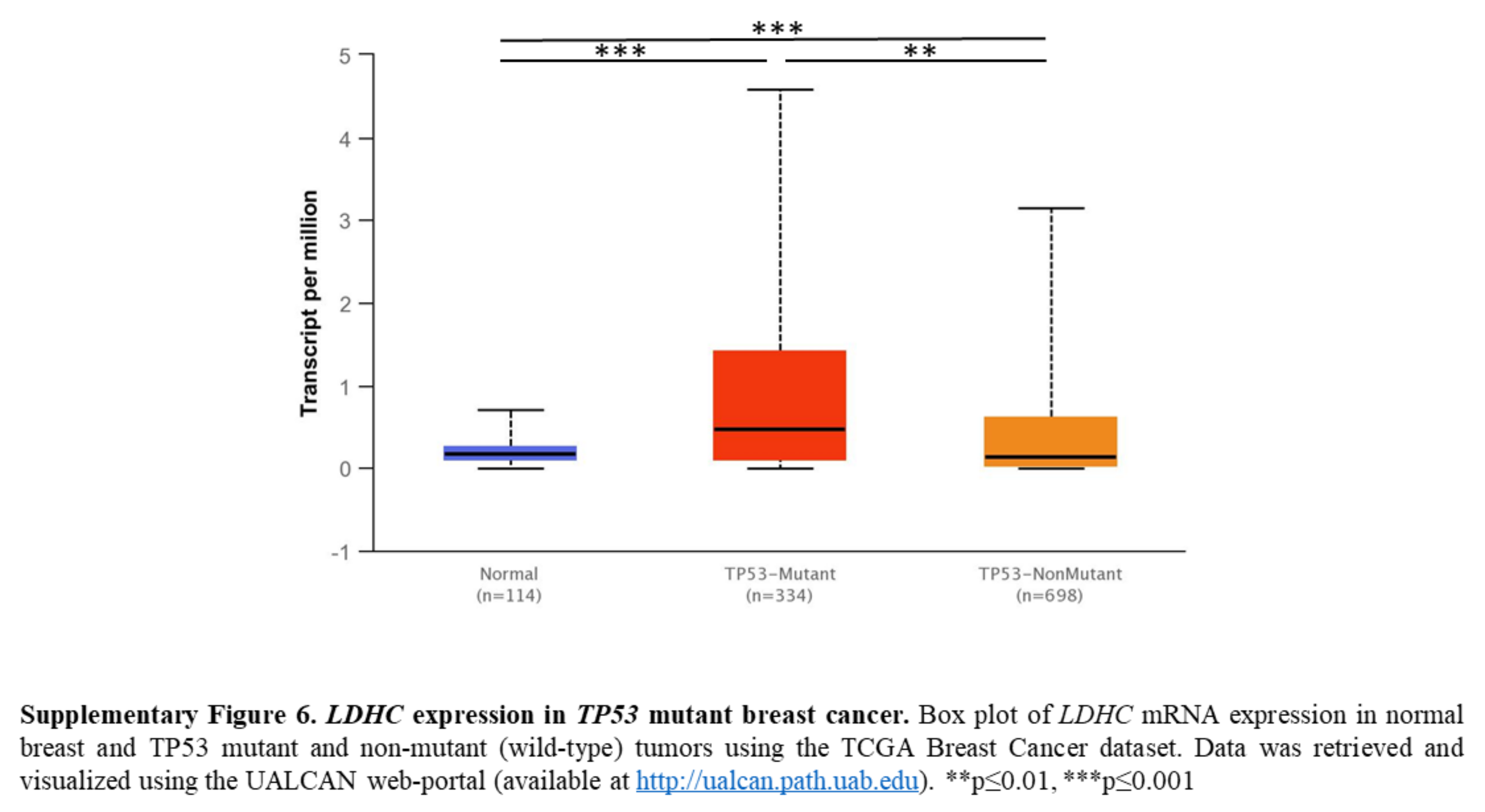

Supplement: Supplementary file 6 — Fig. S6. LDHC expression in TP53 mutant breast cancer. [file MOL2-16-885-s003.png]
