# Supplementary material for: Targeting of lactate dehydrogenase C dysregulates the cell cycle and sensitizes breast cancer cells to DNA damage response targeted therapy
Source: Mol Oncol. 2021 Jun 13;16(4):885–903. doi: 10.1002/1878-0261.13024 (PMC8847988; doi:10.1002/1878-0261.13024)
Supplement: Supplementary file 7 — Table S1. List of antibodies. [file MOL2-16-885-s001.pdf]

Supplementary Table 1. List of antibodies

Antibody	Catalog no.	Manufacturer	Host	Concentration
Acetylated alpha-tubulin (Lys40)	5335	Cell Signaling	Rabbit	1:200 (IF)
Alpha-tubulin	ab52866	Abcam	Rabbit	1:1000 (WB), 1:200 (IF)
ATM (D2E2)	2873	Cell Signaling	Rabbit	1:1000 (WB)
ATR (E1S3S)	13934	Cell Signaling	Rabbit	1:1000 (WB)
Aurora A	14475	Cell Signaling	Rabbit	1:1000 (WB)
Beta-Actin	4970	Cell Signaling	Rabbit	1:1000 (WB)
Beta-tubulin	2128	Cell Signaling	Rabbit	1:1000 (WB), 1:200 (IF)
BubR1	ab28193	Abcam	Sheep	1:500 (WB), 1:100 (IF)
Caspase 3	9662	Cell Signaling	Rabbit	1:1000 (WB)
Cdc25C	4688	Cell Signaling	Rabbit	1:1000 (WB)
CDK6	3136	Cell Signaling	Mouse	1:1000 (WB)
CKS2	ab155078	Abcam	Rabbit	1:1000 (WB)
Cyclin B1	12231	Cell Signaling	Rabbit	1:1000 (WB)
Cyclin D1	2978	Cell Signaling	Rabbit	1:1000 (WB)
Cyclin E2	4132	Cell Signaling	Rabbit	1:1000 (WB)
DNA-PKcs (E6U3A)	38168	Cell Signaling	Rabbit	1:1000 (WB)
LDHC	ab52747	Abcam	Rabbit	1:500 (WB)
Mad2L1	ab97777	Abcam	Rabbit	1:1000 (WB)
MAP1B	ab11266	Abcam	Mouse	1:200 (IF)
Myt1	4282	Cell Signaling	Rabbit	1:1000 (WB)
P16	80772	Cell Signaling	Rabbit	1:1000 (WB)
P18	2896	Cell Signaling	Mouse	1:1000 (WB)
P21	2947	Cell Signaling	Rabbit	1:1000 (WB)

P27	3686	Cell Signaling	Rabbit	1:1000 (WB)
P53	2527	Cell Signaling	Rabbit	1:1000 (WB)
Phalloidin-Alexa Fluor 568	A12380	Thermo Fisher		1:100 (IF)
Phospho-BRCA1 (Ser1524)	9009	Cell Signaling	Rabbit	1:1000 (WB)
Phospho-gamma H2AX (S139)	ab11174	Abcam	Rabbit	1:1000 (WB), 1:200 (IF)
Phospho-cdc2 (Tyr15)	4539	Cell Signaling	Rabbit	1:1000 (WB)
Phospho-Cdc25C (Ser216)	4901	Cell Signaling	Rabbit	1:1000 (WB)
Phospho-Chk1 (Ser345)	2348	Cell Signaling	Rabbit	1:1000 (WB)
Phospho-Chk2 (Thr68)	2197	Cell Signaling	Rabbit	1:1000 (WB)
Phospho-Myt1 (Ser83)	4281	Cell Signaling	Rabbit	1:1000 (WB)
Phospho-Wee1 (Ser642)	4910	Cell Signaling	Rabbit	1:1000 (WB)
Wee1	13084	Cell Signaling	Rabbit	1:1000 (WB)

WB, western blotting; IF, immunofluorescence
