# Supplementary material for: Targeting of lactate dehydrogenase C dysregulates the cell cycle and sensitizes breast cancer cells to DNA damage response targeted therapy
Source: Mol Oncol. 2021 Jun 13;16(4):885–903. doi: 10.1002/1878-0261.13024 (PMC8847988; doi:10.1002/1878-0261.13024)
Supplement: Supplementary file 8 — Table S2. Differentially expressed cell cycle genes. [file MOL2-16-885-s005.pdf]

Supplementary Table 2. Differentially expressed cell cycle genes. Differentially expressed genes after LDHC silencing in MDA-MB-468 cells as determined by the Cell Cycle RT2 Profiler qPCR array.

Gene Symbol	Fold Regulation
AURKA	4.60
AURKB	2.80
BIRC5	4.28
BRCA1	2.24
BRCA2	2.83
CCNA2	4.03
CCNB1	3.27
CCNB2	2.42
CCND3	2.77
CCNF	4.15
CDC20	3.29
CDC25A	2.90
CDC25C	3.50
CDC6	2.32
CDK1	3.40
CDK2	2.29
CDKN3	2.21
CHEK2	2.39
CKS2	2.05
E2F1	2.51
GTSE1	3.58
KPNA2	2.36
MAD2L1	2.50
MKI67	4.12
RAD51	2.24
STMN1	2.06
