# Supplementary material for: Targeting of lactate dehydrogenase C dysregulates the cell cycle and sensitizes breast cancer cells to DNA damage response targeted therapy
Source: Mol Oncol. 2021 Jun 13;16(4):885–903. doi: 10.1002/1878-0261.13024 (PMC8847988; doi:10.1002/1878-0261.13024)
Supplement: Supplementary file 9 — Table S3. Summary of observed characteristics in LDHC‐silenced breast cell lines. [file MOL2-16-885-s009.pdf]

Supplementary Table 3. Summary of observed characteristics in LDHC-silenced breast cell lines

	MDA-MB-468	BT-549	MDA-MB-231	HCC-1500
Parental cell properties				
Molecular subtype	Basal-like	Basal-like	Basal-like	Luminal A
BRCA	Wild-type	Wild-type	Wild-type	Wild-type
p53	mutant	mutant	mutant	negative
Rb	negative	negative	positive	positive
LDHC silencing phenotypes				
Giant cells	Increase	Increase	Increase	Increase
Polyploidy	Increase	Increase	**low baseline level, no change**	**low baseline level, no change**
DNA damage	Increase	Increase	Increase	Increase
Apoptosis	Increase	Increase	Increase	Increase
Long-term survival/Clonogenicity	Decrease	Decrease	Decrease	Decrease
Microtubule instability	Increase	Increase	Increase	Increase
Cell population in G1	Decrease	Decrease	Decrease	Decrease
Cell population in G2/M	Increase	Increase	Increase	Increase
Mitotic slippage	Increase	Increase	**ND**	**ND**
Cell cycle arrest	Increase	Increase	**ND**	**ND**
Senescence	No change	No change	No change	**Increase**
Sensitivity to Cisplatin	Increase	Increase	Increase	**No change**
Sensitivity to Olaparib	Increase	Increase	Increase	Increase
